# Transcriptome Analyses Reveal Candidate Pod Shattering-Associated Genes Involved in the Pod Ventral Sutures of Common Vetch (*Vicia sativa* L.)

**DOI:** 10.3389/fpls.2017.00649

**Published:** 2017-04-27

**Authors:** Rui Dong, Deke Dong, Dong Luo, Qiang Zhou, Xutian Chai, Jiyu Zhang, Wengang Xie, Wenxian Liu, Yang Dong, Yanrong Wang, Zhipeng Liu

**Affiliations:** ^1^State Key Laboratory of Grassland Agro-ecosystems, College of Pastoral Agriculture Science and Technology, Lanzhou UniversityLanzhou, China; ^2^State Key Laboratory of Systematic and Evolutionary Botany, Institute of Botany, Chinese Academy of SciencesBeijing, China

**Keywords:** *Vicia sativa* L., transcriptome, pod ventral suture, pod shattering, cell wall hydrolases

## Abstract

The seed dispersion caused by pod shattering is a form of propagation used by many wild species. Loss of seeds from pod shattering is frequent in the common vetch (*Vicia sativa* L.), an important self-pollinating annual forage legume. However, pod shattering is one of the most important defects that limits the reproduction of the vetch in the field and the usage as a leguminous forage crop. To better understand the vetch pod shattering mechanism, we used high-throughput RNA sequencing to assess the global changes in the transcriptomes of the pod ventral sutures of shattering-susceptible and shattering-resistant vetch accessions screened from 541 vetch germplasms. A total of 1,285 significantly differentially expressed unigenes (DEGs) were detected, including 575 up-regulated unigenes and 710 down-regulated unigenes. Analyses of Gene Ontology and KEGG metabolic enrichment pathways of 1,285 DEGs indicated that 22 DEGs encoding cell wall modifications and hydrolases associated with pod shattering were highly expressed in shattering-susceptible accessions. These genes were mainly enriched in “hydrolase activity,” “cytoplasm,” and “carbohydrate metabolic process” systems. These cell wall modifications and hydrolases genes included β-glucosidase and endo-polygalacturonase, which work together to break down the glycosidic bonds of pectin and cellulose, and to promote the dissolution and disappearance of the cell wall in the ventral suture of the pod and make the pod more susceptible to shattering. We demonstrated the differences in gene transcription levels between the shattering-susceptible and shattering-resistant vetch accessions for the first time and our results provided valuable information for the identifying and characterizing of pod shattering regulation networks in vetch. This information may facilitate the future identification of pod shattering-related genes and their underlying molecular mechanisms in the common vetch.

## Introduction

In nature, seed dispersion or fruit dehiscence is an essential process in the proliferation of wild plants (Funatsuki et al., 2014). This process can be used to ensure adequate growth space for the progeny of plants in nature (Funatsuki et al., 2014). This agronomic trait exists in most Leguminosae, Gramineae, and Brassicaceae crops, such as common vetch (*Vicia sativa* L.) (Abd El-Moneim, 1993), soybeans (*Glycine max* L.) (Christiansen et al., 2002), rice (*Oryza sativa* L.) (Dong et al., 2014), thale cress (*Arabidopsis thaliana* L.) (Dong et al., 2014) and oilseed rape (*Brassica napus* L.) (del Carmen Rodríguez-Gacio et al., 2004). However, since the domestication of plants began, humans have attempted to breed non-shattering crop varieties to reduce seed dispersal, because this agronomical trait leads to a reduction in seed yield (Funatsuki et al., 2006; Dong et al., 2014). Harvesting pod shattering-susceptible soybean varieties in dry weather conditions during the harvest period can lead to seed losses of 50–100% (Bhor et al., 2014). For oilseed rape, average annual seed losses due to pod shattering are ~20–50% of the total seed yield. Therefore, eliminating pod shattering would in theory produce an equivalent increase in harvested yield (Squires et al., 2003; Østergaard et al., 2006; Raman et al., 2014). In addition, seeds dispersed by pod shattering can persist in the soil seed bank for up to 10 years, thus leading to the appearance of a crop as a weed in subsequent crop growing seasons (Pekrun et al., 1997). Therefore, resistance to pod shattering has been preferentially selected for during domestication as the most important domestication trait (Funatsuki et al., 2014).

In plants, seed dispersal or pod shattering has been revealed to be associated primarily with the separation, elimination or modification of the dehiscence zone tissue cells (Dong et al., 2014). Adhesion between cells is a fundamental feature of plant growth and development and is an important factor in maintaining the mechanical strength of plants. In dicotyledonous plants, the adherent region is occupied by a network of various pectin polymers. When the plant cells separate, the pectin polymer network of the adherent cells decomposes in the local tissue. This phenomenon occurs during pod shattering, seed dispersal and fruit ripening (Jarvis et al., 2003). Enzymatic cleavage of the galacturonan chain of pectin is an effective method of solubilizing pectin, and usually separates dicotyledonous cells (Zhang et al., 2000; Jarvis et al., 2003). Polygalacturonases have been found to be involved in the hydrolysis of pectin in many stages of plant development, particularly in tissues that require cell separation (Hadfield and Bennett, 1998). Previous studies have shown that some specific genes are expressed during the plant abscission process. The products of these genes include polygalacturonase, β(1,4)-D-glucanase and other enzymes (Hong and Tucker, 1998). In addition, most of these enzymes belong to the isotype specific to the abscission zone. The network of cellulose and hemicellulose is also a major component of the plant primary cell wall. In *Arabidopsis* and other dicotyledons, xyloglucan is the major cell wall hemicellulose (Lashbrook and Cai, 2008). Other cell wall modification enzymes also play an important role in the breakdown of the cell wall and the degradation of the dehiscence zone, such as endo-polygalacturonase, pectin methylesterases and β-galactosidase (Christiansen et al., 2002; Tavarini et al., 2009; Zhang et al., 2012). One of the most notable enzymes is the endo-galacturonase *RDPG1*, which has been shown to promote the breakdown of the middle lamella (Christiansen et al., 2002). Several genes encoding transcription factors required for silique development and shattering in *Arabidopsis* have been extensively studied at the molecular level (Zhang et al., 2016).

In recent years, several soybean pod shattering studies have obtained exciting results. Researchers have found a key new cellular feature in the abscission layer of the shattering-resistant soybean pod ventral suture, called fiber cap cells (FCC) (Dong et al., 2014). Increased expression of the *GmSHAT1-5* gene leads to increased thickening of the secondary cell wall of FCC, thus preventing pod shattering in the domesticated soybean. Furthermore, removing repressive elements enhances the expression of *GmSHAT1-5* in the FCC and promotes the over-deposition of the secondary cell wall (Dong et al., 2014). Another functional gene, *pdh1*, has been found to be highly expressed in the lignin-rich internal sclerenchyma of soybean pod walls, particularly at the initial stage of lignin deposition (Funatsuki et al., 2014). A comparison of near isogenic lines has shown that *pdh1* promotes pod shattering by increasing the torsion force of the dry pod wall, which is a driving force of pod shattering in dry climates (Funatsuki et al., 2014).

The common vetch is an important self-pollinating annual forage legume (Chung et al., 2013; Liu et al., 2013a; Dong et al., 2016a). Owing to its broad environmental adaptation and high nutritional value, it is used for hay production, pasture, silage, and green manure (Sullivan and Diver, 2003; Liu et al., 2014; Kim et al., 2015; Dong et al., 2016a). It is widely planted in Turkey, Australia, New Zealand, the Qinghai-Tibetan plateau of China and other parts of the world (Dong et al., 2016a). In Turkey, the planted area of the common vetch has reached 579,684 ha (Firincioglu, 2012). However, the shattering of mature vetch pods is one of the most important defects limiting its utilization (Abd El-Moneim, 1993). The results of hybridization of shattering-susceptible and shattering-resistant accessions have indicated that the F2 populations are separated according to a 3:1 ratio (susceptible: resistant) (Abd El-Moneim, 1993). In addition, the research regarding vetch pod shattering is still at a preliminary level of phenotypic observation (van de Wouw et al., 2003). Next-generation sequencing (NGS) is currently the preferred method for understanding gene expression in non-model plants at different developmental stages on a transcriptome-wide scale (Liu et al., 2016). By comparing the transcriptomes of the pod ventral sutures of shattering-susceptible and shattering-resistant accessions screened from 541 vetch accessions, the present study provides a good understanding of the genetic basis of pod shattering in the common vetch, which exhibits different dehiscence zone structures from those of Arabidopsis and soybeans. This study provides additional genetic information for the study of plant pod shattering.

## Materials and methods

### Plant materials and sample collection

A total of four common vetch accessions were used in this study: 92, 135, 257, and 392. The four accessions were screened from 541 vetch accessions by evaluation of the pod shattering index in three consecutive years (2012–2014). Seeds of these four vetch accessions were provided by the National Plant Germplasm System of the United States and Lanzhou University, China. The seeds were sown in the Yuzhong Experimental Station of Lanzhou University (N 35°57′, E 104°09′, 1,720 m above sea level), Lanzhou, China. The mean annual precipitation in this area is 350 mm, and the mean annual temperature is 6.7°C.

**Figure 1:**
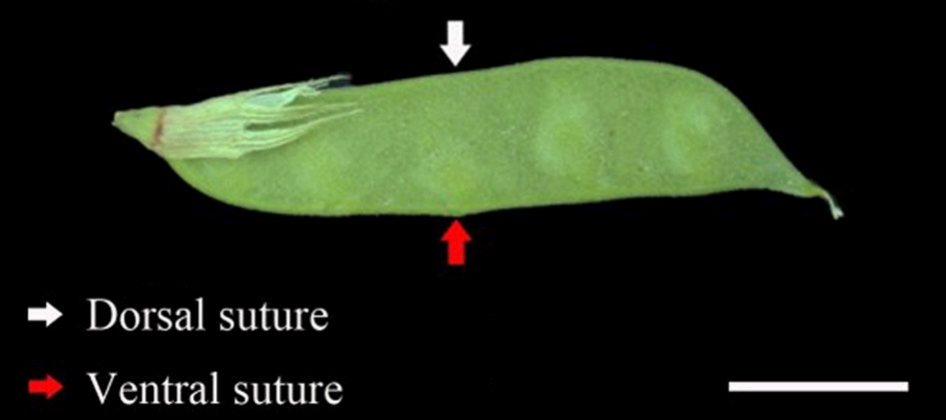
**The developmental phenotype of the sampled pods**. The red arrows indicate the pod ventral suture. The white arrows indicate the pod dorsal suture. Scale bar, 1 cm.

At flowering time, 400 flowers were tagged for each accession on different plants. The pod ventral sutures of the four accessions were the samples used in this study (Figure 1). Pod ventral sutures were selected as the material for the study of pod shattering because our previous study has found that vetch pod shattering is initiated in the pod ventral suture.

The sampling time was the period of time required for the formation of the dehiscence zone in the pod ventral sutures. Because the growth periods of the different vetch accessions were not consistent, the collection time of the samples from the four accessions was different. The detailed collection times are shown in Table S1. At the time of sampling, the seed size was approximately one-third of the final size, and the pod length and width were 80–90% those of the mature yellow pod. The pods were collected and placed on ice, and the pod ventral sutures were manually removed. The freshly removed pod ventral suture samples were immediately frozen in liquid nitrogen and stored at −80°C for RNA isolation.

### Pod shattering index evaluation

The pod shattering rate and pod shattering mechanical force were used to evaluate the pod shattering index of the four accessions. Under field conditions (without artificial disturbance), the number of shattering pods of each accession was counted out of 100 pods after pod maturity in three consecutive years (2012–2014). Under laboratory conditions, 100 mature pods were collected for each accession, air-dried at room temperature for 2 weeks, and kept in an oven at 37°C for 24 h, and the minimum mechanical force necessary to break the pods from the different accessions into two parts was measured by using a digital mechanical force gauge (HANDPI, China) (Dong et al., 2014, 2016b). All statistical analyses were performed on three replicates.

### Polygalacturonase and cellulase activity assays

The activity of polygalacturonase and cellulase (β-1,3;1,4-glucanase) in the pod ventral sutures of the four accessions was determined. Polygalacturonase activity was determined using the method described by Lohani et al. (2004) and Gross (1982). Cellulase (β-1,3;1,4-glucanase) activity was measured according to the reducing groups released from carboxymethyl cellulose (CMC), as described by Wu et al. (2008).

### Pod ventral suture structure

The pod ventral suture structures from the four different accessions were frozen during the sampling period, and frozen sections were then observed. The pod samples were fixed with FAA fixation solution for 24 h and embedded in OCT (Sakura, USA) embedding agent, and 5 μm sections were then dissected using a cryostat (LEICA CM3050S, Germany) and analyzed using an OLYMPUS microscope (OLYMPUS BX51, Japan) (Kawamoto, 2003).

### RNA extraction and library construction for transcriptome analysis

Total RNA of the four accessions was extracted using Trizol reagent according to the manufacturer's instructions (Invitrogen, Carlsbad, CA, USA). The RNA concentrations were determined using a Qubit® RNA Assay Kit and a Qubit®2.0 Fluorometer (Life Technologies, CA, USA), and the RNA integrity was assessed using an RNA Nano 6000 Assay Kit with an Agilent Bioanalyzer 2100 system (Agilent Technologies, CA, USA). Poly(A)-containing mRNAs were purified from the total RNA with oligo (dT) magnetic beads (Illumina, San Diego, CA). Then, mRNA random fragmentation, cDNA synthesis, purification and PCR amplification were performed according to the Illumina RNA-Seq protocol. The four cDNA libraries were sequenced with a read length of 194 bp on a HiSeq 2000 system with a paired-end module at Biomarker Co. Ltd. (Beijing, China).

### Sequence filtering, *de novo* assembly and functional annotation

The raw sequencing reads were cleaned by using the FASTX toolkit. A *de novo* transcriptome assembly of all the reads was performed using the Trinity Program (Grabherr et al., 2011). According to the Trinity assembly results, all unigenes were annotated on the basis of a BLASTx alignment, with 10^−5^ as the *E*-value cut off point, to sequence comparisons with public databases, including the NCBI non-redundant protein sequences (Nr), Gene Ontology (GO), Clusters of Orthologous Groups of proteins (COGs), Kyoto Encyclopedia of Genes and Genomes (KEGG), and Swiss-Prot protein databases. The amino acid sequences of the unigenes were predicted and compared against the Pfam protein database using HMMER 3.0 (*E*-value ≤ 1e^−10^) to obtain unigene domain/family annotation information. The results that aligned best against the five protein databases were used to determine the sequence directions of the unigenes. The BLAST hits were analyzed using Blast2GO software to obtain GO terms to retrieve molecular function, biological process and cellular component descriptions (Liu et al., 2013b; Calzadilla et al., 2016). The GO annotation functional classifications were determined using WEGO software for the distribution of gene functions (Ye et al., 2006).

### Differential expression analysis

The reads obtained from the sequencing of each sample were compared against the unigene library by using Bowtie, and the expression was calculated according to the alignment results and RSEM (Langmead et al., 2009; Li and Dewey, 2011; Langmead and Salzberg, 2012). The differential expression analysis was performed using the MARS method in the DEGseq 2010 R package (Anders and Huber, 2010; Wang et al., 2010). The analysis was performed using Benjamini and Hochberg correction of the *p*-values and finally using the corrected *p*-value and the FDR (False Discovery Rate) to reduce false positives (Benjamini and Hochberg, 1995; Sivankalyani et al., 2016). The *p*-value was adjusted using the *q*-value (Shannon et al., 2003). A *q*-value < 0.005 and an absolute value of the log2 (fold change) >1 were used as thresholds for determining the significant DEGs. The DEG expression patterns were clustered using STEM software (*p*-value ≤ 0.05). A GO enrichment analysis was performed for all the DEGs using topGO software and agriGO, and KOBAS 2.0 was used to carry out the KEGG pathway enrichment analysis (Liu et al., 2016).

### Quantitative real-time PCR analysis

A portion of the total RNA of the four samples used for the RNA-Seq analysis was used to make cDNA for qRT-PCR analysis. An M-MuLV First Strand cDNA Synthesis Kit (TaKaRa Biotechnology, Dalian, China) was used according to the manufacturer's instructions to generate cDNA from 1 μg of total RNA from each sample. The cDNA samples were diluted to a concentration of 1 ng/ml. Quantitative qRT-PCR was performed using an iQ 5 Multicolor Real-time PCR Detection system (Bio-Rad, USA) and Power SYBR Green Master Mix (Applied Biosystems), by following the manufacturer's protocol (TaKaRa SYBR Premix Ex Taq II Kit, Dalian, China). Unigene 68614, with unknown function, was selected as an internal control gene, owing to its relatively stable expression in the common vetch transcription profiles (Liu et al., 2013a). Specific primers for qRT-PCR were designed by using Primer Express software and are shown in Table S2. The qRT-PCR analysis of each sample was performed in triplicate. The expression levels of each unigene were normalized to that of unigene 68614, and the relative gene expression levels were calculated using the 2^−ΔΔ^Ct method.

## Results

### Pod shattering index

**Figure 2:**
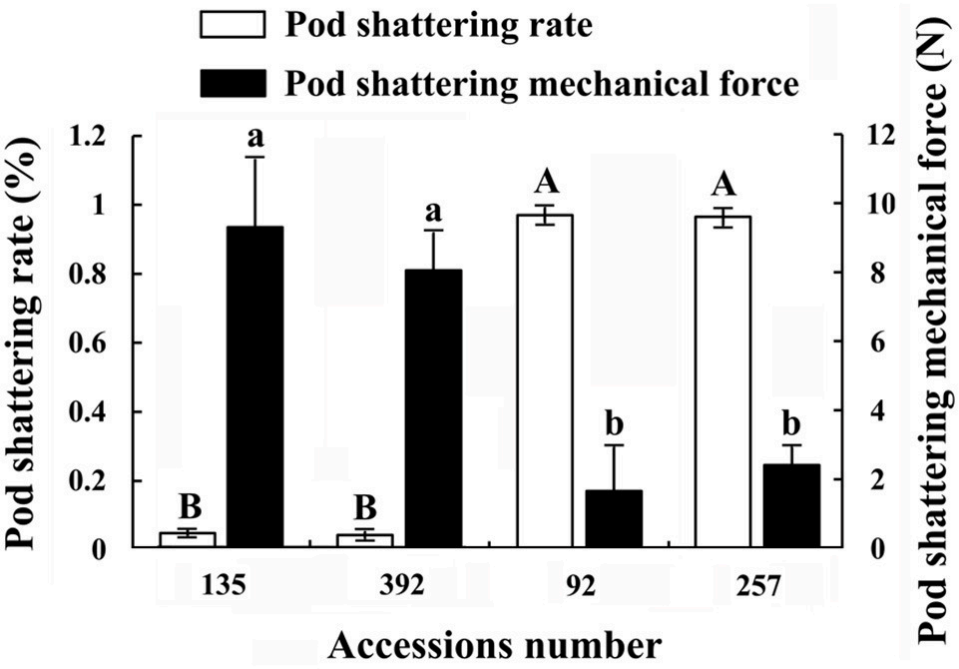
**Pod shattering rate and pod shattering mechanical force of four common vetch accessions**. Pod shattering rate refers to the rate of pod shattering in the field (without artificial disturbance). Three replicates, each with 100 pods, were observed. Values represent mean ± SE. Pod shattering mechanical force refers to the minimum mechanical force necessary to break the pods of different accessions into two parts. This experiment was repeated three times, with 100 pods measured each time. Values represent mean ± SE. Different uppercase and lowercase letters indicate significant differences among the different accessions at *p* < 0.01 according to Tukey's test.

**Figure 3:**
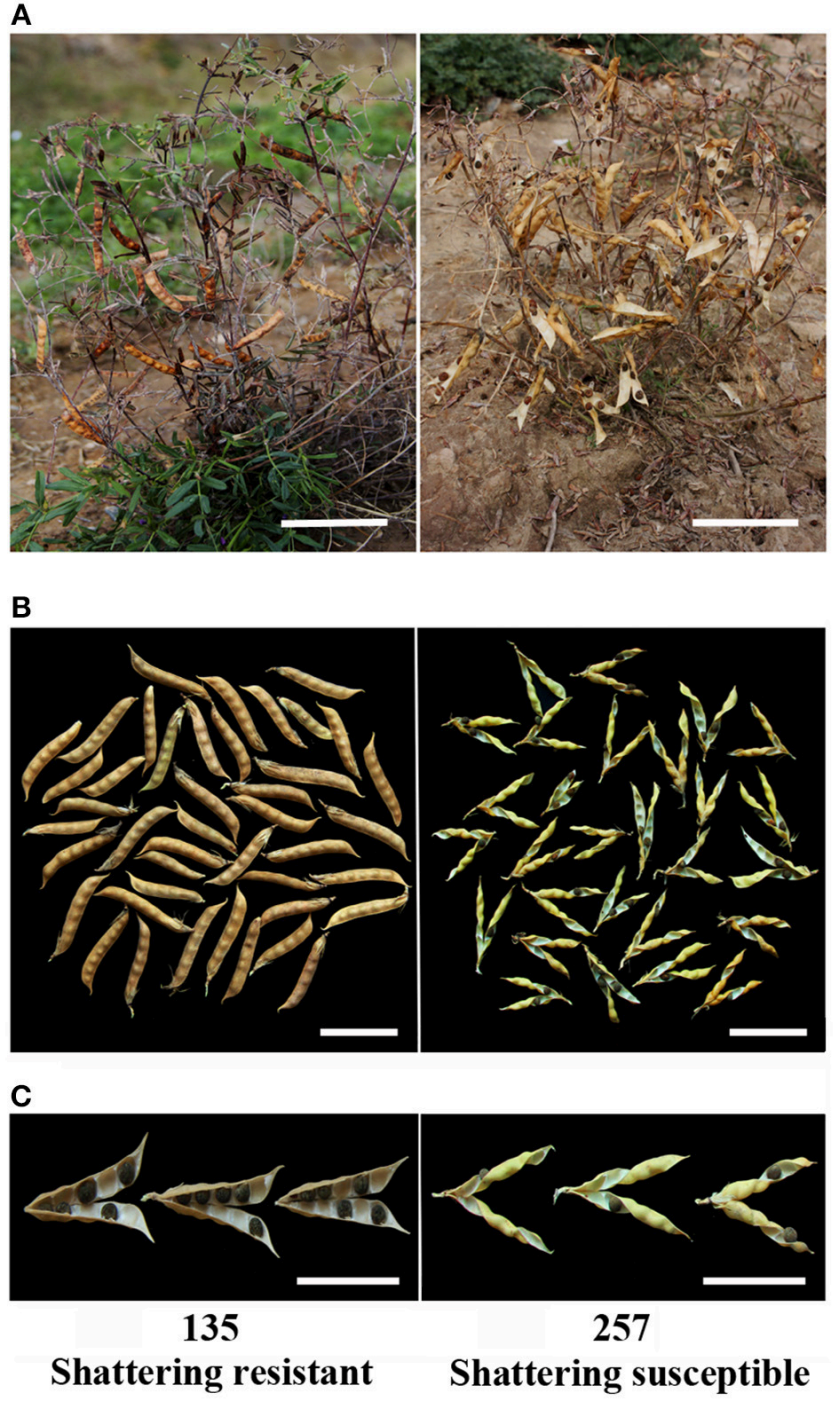
**Pods from two types of pod shattering common vetch accessions. (A)** No. 135 (shattering resistant) and No. 257 (shattering susceptible) accessions, and dehisced pods and shed seeds of the No. 257 accession in the field. **(B)** Dried pods of the No. 135 (left) and No. 257 (right) accessions at ambient humidity (~40% relative humidity). **(C)** Pod wall torsion of No. 135 (after drying, the pods did not shatter and were opened manually) and No. 257 (natural shattering) accessions at 20% relative humidity, dehisced after natural drying. Scale bars in **(A)**, 10 cm; **(B)**, 5 cm; **(C)**, 5 cm.

In this study, the pod shattering rate and pod shattering mechanical force of accessions Nos. 92, 257, 135, and 392 showed significant differences (Figure 2). The results of the pod shattering rate analysis confirmed that Nos. 92 and 257 were pod shattering-susceptible accessions, with pod shattering rates higher than 95%, whereas Nos. 135 and 392 were pod shattering-resistant accessions, with pod shattering rates lower than 5% (Figure 2). Furthermore, the average pod shattering mechanical force of Nos. 135 and 392 was approximately four times that of Nos. 92 and 257 (Figure 2). Figure 3 shows more details of pod shattering in the shattering-susceptible and shattering-resistant accessions under field and low humidity conditions.

### Polygalacturonase and cellulase activity of pod ventral sutures

**Figure 4:**
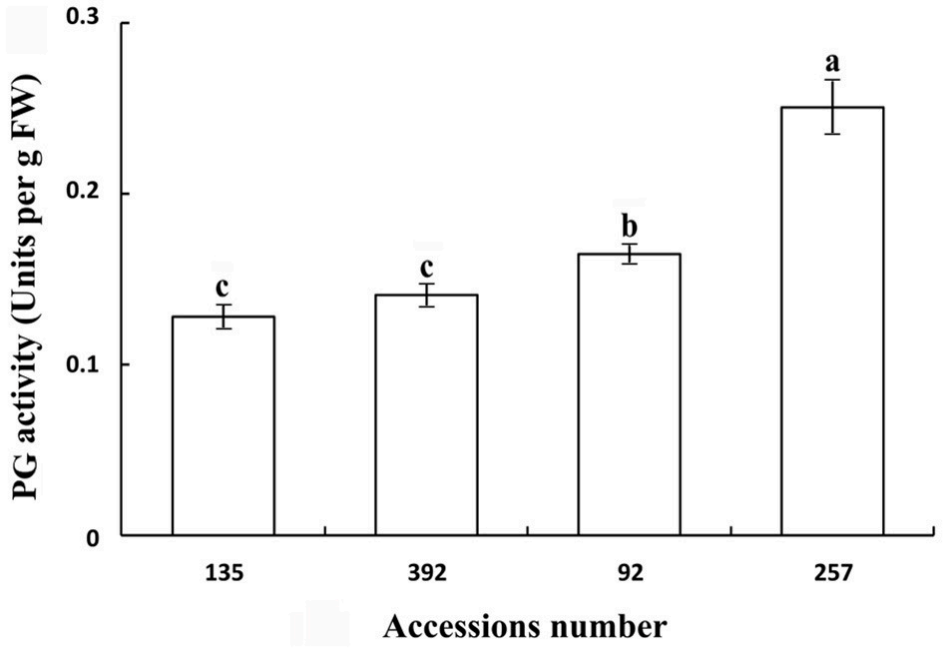
**Polygalacturonase activity in pod ventral sutures of four common vetch accessions**. One unit of polygalacturonase activity is the amount that catalyses the formation of 1 μm of reducing groups per min per g of original fresh weight of pod ventral sutures of the common vetch. PG refers to polygalacturonase. Three replicates were performed, each replicate measuring 30 pod ventral sutures. For each replicate, 30 pods of the ventral sutures were collected, and the 30 pod ventral suture samples were mixed for measurement. Values represent mean ± SE. Different lowercase letters indicate significant differences among the different accessions at *p* < 0.01 according to Tukey's test.

**Figure 5:**
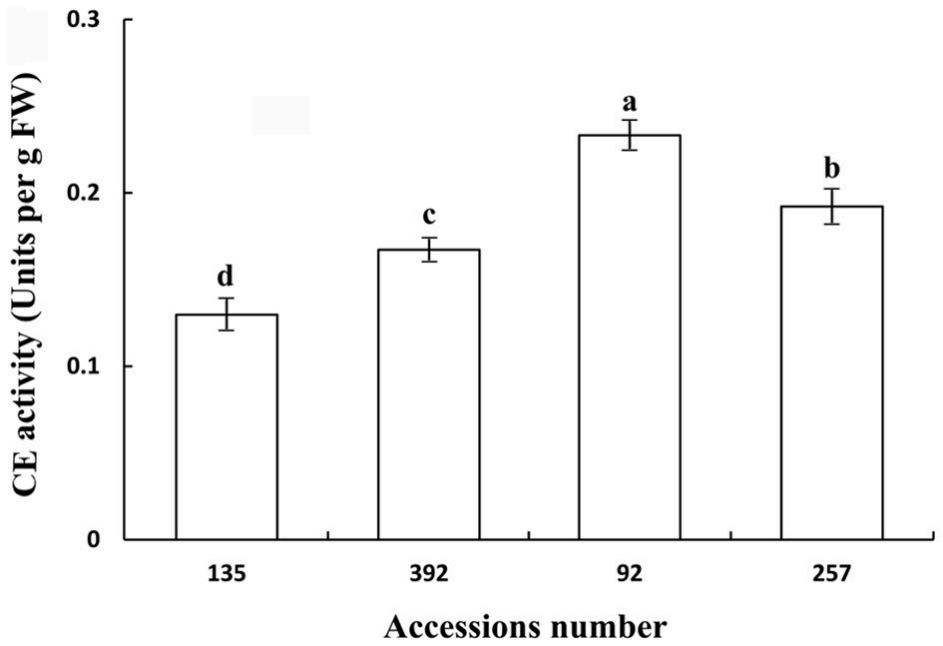
**Cellulase (β-1,3;1,4-glucanase) activity in pod ventral sutures of four common vetch accessions**. One unit of cellulase activity is defined as the amount of the enzyme required to release 1 μm of reducing groups per min per g of original fresh weight of pod ventral sutures of the common vetch. CE refers to cellulase. Three replicates were performed, each replicate measuring 30 pod ventral sutures. For each replicate, 30 pods of the ventral sutures were collected, and the 30 pod ventral suture samples were mixed for measurement. Values represent mean ± SE. Different lowercase letters indicate significant differences among the different accessions at *p* < 0.01 according to Tukey's test.

Polygalacturonase and cellulose (β-1,3;1,4-glucanase) are important hydrolases in the cell wall. The results showed that the activities of polygalacturonase and cellulase (β-1,3;1,4-glucanase) were significantly higher in the pod ventral sutures of the shattering-susceptible accessions than in those of the shattering-resistant accessions (Figures 4, 5). The polygalacturonase activity was highest in accession 257 and lowest in accession 135. The cellulase activity was highest in accession 92 and lowest in accession 135.

### Pod ventral suture structure

**Figure 6:**
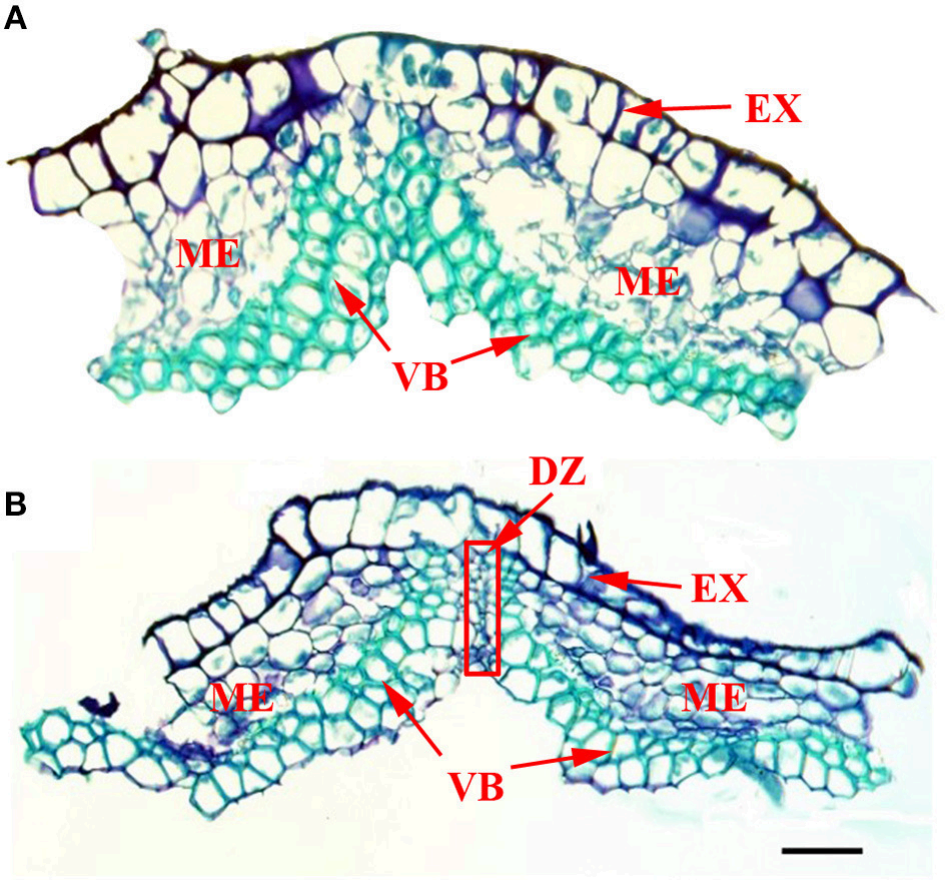
**Frozen cross sections of manually removed pod ventral sutures of the No. 135 and 257 accessions**. At the time of sampling, the seed size was approximately one-third of the final size, and the pod length and width were 80–90% those of the mature yellow pod. **(A)** The structure of a pod ventral suture from the No. 135 accession. The results showed a complete VB structure and no DZ. **(B)** The structure of a pod ventral suture from the No. 257 accession. The results showed a VB structure that was divided into two parts by the DZ. DZ located at the junctions between two VB valves. The red frame in picture B was the DZ. DZ, Dehiscence zone. VB, Vascular bundle; EX, Exocarp; ME, Mesocarp; Scale bar, 100 μm.

Figure 6 shows the structure of pod ventral suture samples that were manually removed. Cross sections of pod ventral sutures of the shattering-susceptible and shattering-resistant accessions showed that both had vascular bundle structures in the pod ventral sutures (Figure 6). In the pod shattering-susceptible accessions, the vascular bundles were separated into two sections by a dehiscence zone (Figure 6B), whereas the pod shattering-resistant accessions did not have a dehiscence zone, and the vascular bundles in these pods were not separated (Figure 6A).

### Transcriptome sequencing and *de novo* assembly

RNA samples from the pod ventral sutures of the four accessions were used to construct cDNA libraries. A total of 145,891,066 raw reads were ultimately obtained from the four libraries. After a stringent quality check and data filtering, a total of 104,234,410 reads and 26.2 GBase of data were obtained (Table 1). The Q20 values of all four samples were higher than 98.5%. Figure S1 shows the sequencing results in more detail. A total of 496,337 contigs (≥100 bp) were obtained, wherein the N50 of the contigs was 126 bp, and the average size of the contigs was 71 bp. Figure S2 shows the distribution of the contig assembly. The total contigs were assembled into 70,742 unigenes, and 47,921 unigenes were identified for No. 92, 44,922 for No. 135, 45,325 for No. 257 and 43,603 for No. 392 (Table 1). The N50 of these unigenes was 1,309, and the mean length was 772 bp. Of the 70,742 unigenes, 39,339 unigenes (55.61%) were between 200 and 500 bp, 14,539 unigenes (20.55%) were between 501 and 1,000 bp, and 16,861 unigenes (23.84%) were longer than 1,000 bp (Figure S3). The reads of this study have been deposited in the NCBI SRA database (SRX2400610).

**Table 1:** **Summary of the transcriptome data**.

**Sample**	**Total raw reads**	**Total clean reads**	**Total clean nucleotides (nt)**	**Q20 (%)**	**Unigenes**
135	43,293,509	27,925,411	7.0 G	98.64	44,922
392	41,401,651	26,212,623	6.6 G	98.63	43,603
92	31,179,196	24,103,592	6.1 G	98.56	47,921
257	30,016,710	25,992,784	6.5 G	98.65	45,325
Total	145,891,066	104,234,410	26.2 G		70,742

### Functional annotation and classification

The results indicated that 42,074 (59.48%) unigenes were annotated and aligned to known proteins in the Nr database and that 26,134 (36.94%) were aligned to sequences in the Swiss-Prot database (Table S3). In addition, 3,691 (5.22%) unigenes were annotated in all seven databases, and 46,763 (66.10%) unigenes were annotated in at least one database (Table S3). Further analysis of the matching sequences revealed that 32.82% of the sequences had the closest match to sequences from *Medicago truncatula* (Figure S4).

GO assignments were used to classify the functions of the predicted pod ventral suture genes in vetch. In this study, 23,533 unigenes were assigned to a total of 51 GO functional groups and 22,383 GO annotations, which fell into the three main categories (Figure S5). A total of 37,193 unigenes were distributed under the cellular component category, 28,411 unigenes were distributed under the molecular function category, and 59,076 unigenes were distributed under the biological process category.

All the unigenes were assigned to COGs for further functional prediction and classification. In total, 17,281 unigenes were assigned to 25 COG classifications (Figure S6). Among the 25 COG categories, the class “general function prediction only” (16.69%) represented the largest group and was followed by “replication, recombination and repair” (8.57%) and “transcription” (8.05%).

To explore the pathways that involved these annotated genes, the unigenes were also compared against the KEGG database. As a result, a total of 15,571 annotated unigenes had significant matches with 16,070 hits in the KEGG database and were assigned to 127 KEGG pathways (Table S4). Of these unigenes, 10,453 were assigned to the metabolic pathway, which is the largest group of the five categories classified by KEGG (Figure S7A). The 10,453 metabolic pathway genes were further classified into 18 subcategories (Figure S7B), most of which mapped to carbohydrate metabolism (2,486), translation (1,663) and amino acid metabolism (1,622).

### qRT-PCR verification

**Figure 7:**
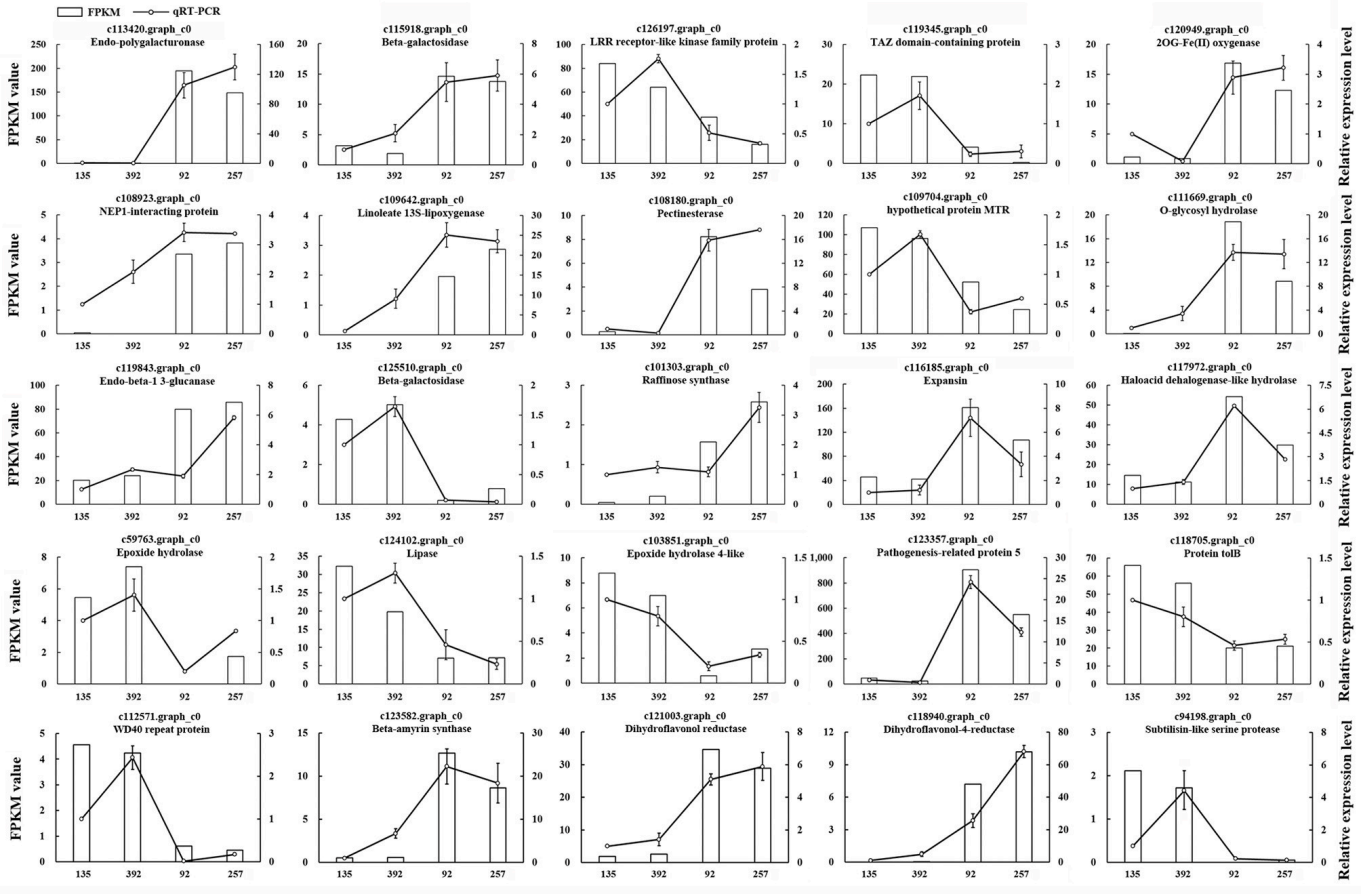
**The expression patterns of 25 randomly selected genes identified by RNA-Seq were validated by qRT-PCR**. White bars indicate the transcript abundance change based on the FPKM values of the RNA-Seq analysis (left y-axis). Lines represented the relative expression levels determined by qRT-PCR (right y-axis). Error bars indicated standard errors of the means (*n* = 3).

To verify the accuracy and reproducibility of the RNA-seq analysis, 25 unigenes were randomly selected for quantitative real-time PCR (qRT-PCR) validation (Table S2). The expression profiles of these 25 unigenes determined by qRT-PCR were consistent with the RNA-seq data (Figure 7). A linear regression analysis showed a significant positive correlation (Pearson correlation coefficient, *r* = 0.87) between the gene expression ratios determined by the two different methods, thus indicating that our RNA-seq data were reliable.

### Analysis of differentially expressed genes (DEGs)

**Figure 8:**
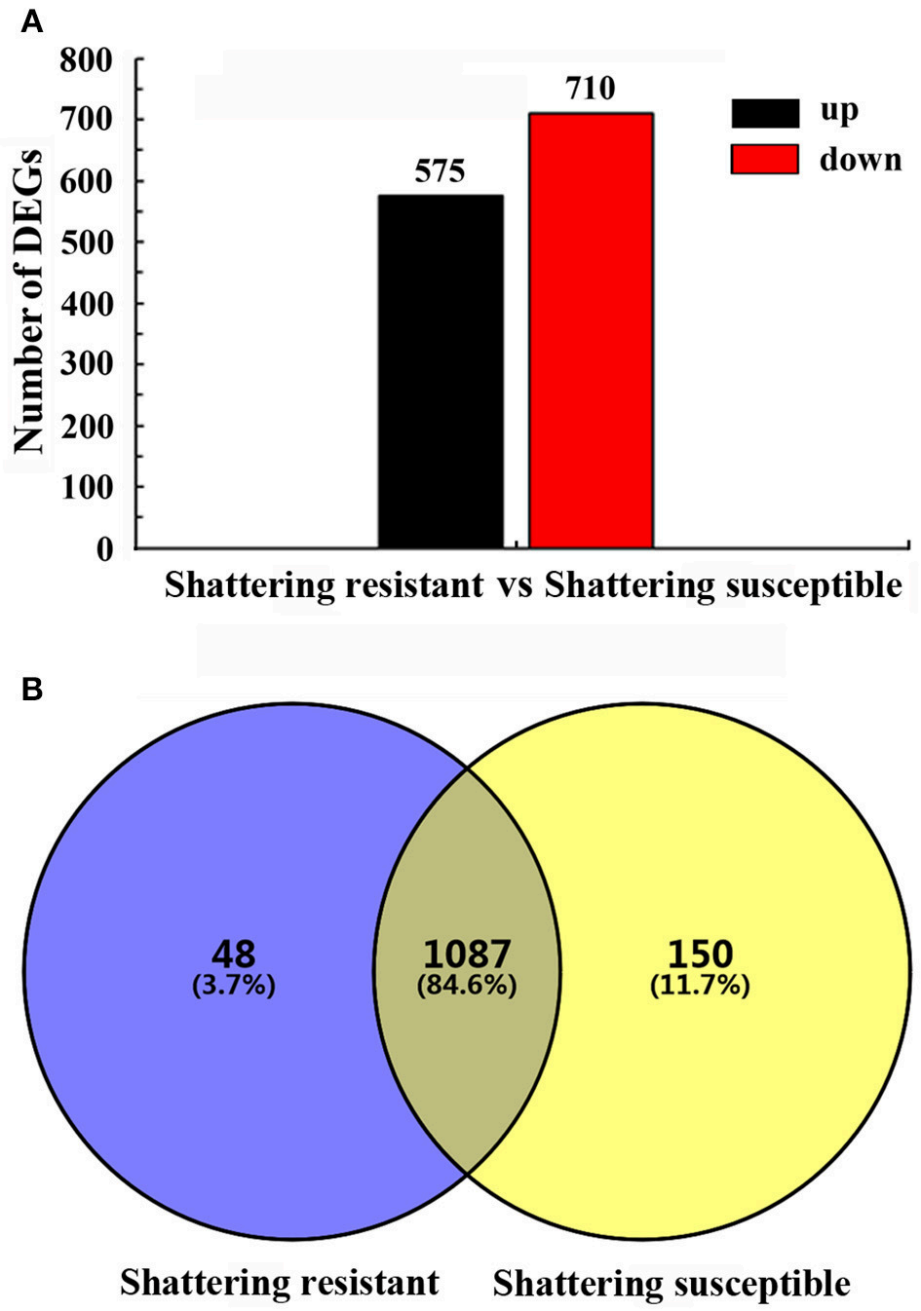
**Summary of the differentially expressed unigenes (DEGs). (A)** The numbers of up- and down-regulated DEGs in shattering-resistant and shattering-susceptible accessions. **(B)** DEGs expressed at the sampling time point between the shattering-resistant and shattering-susceptible accessions.

**Figure 9:**
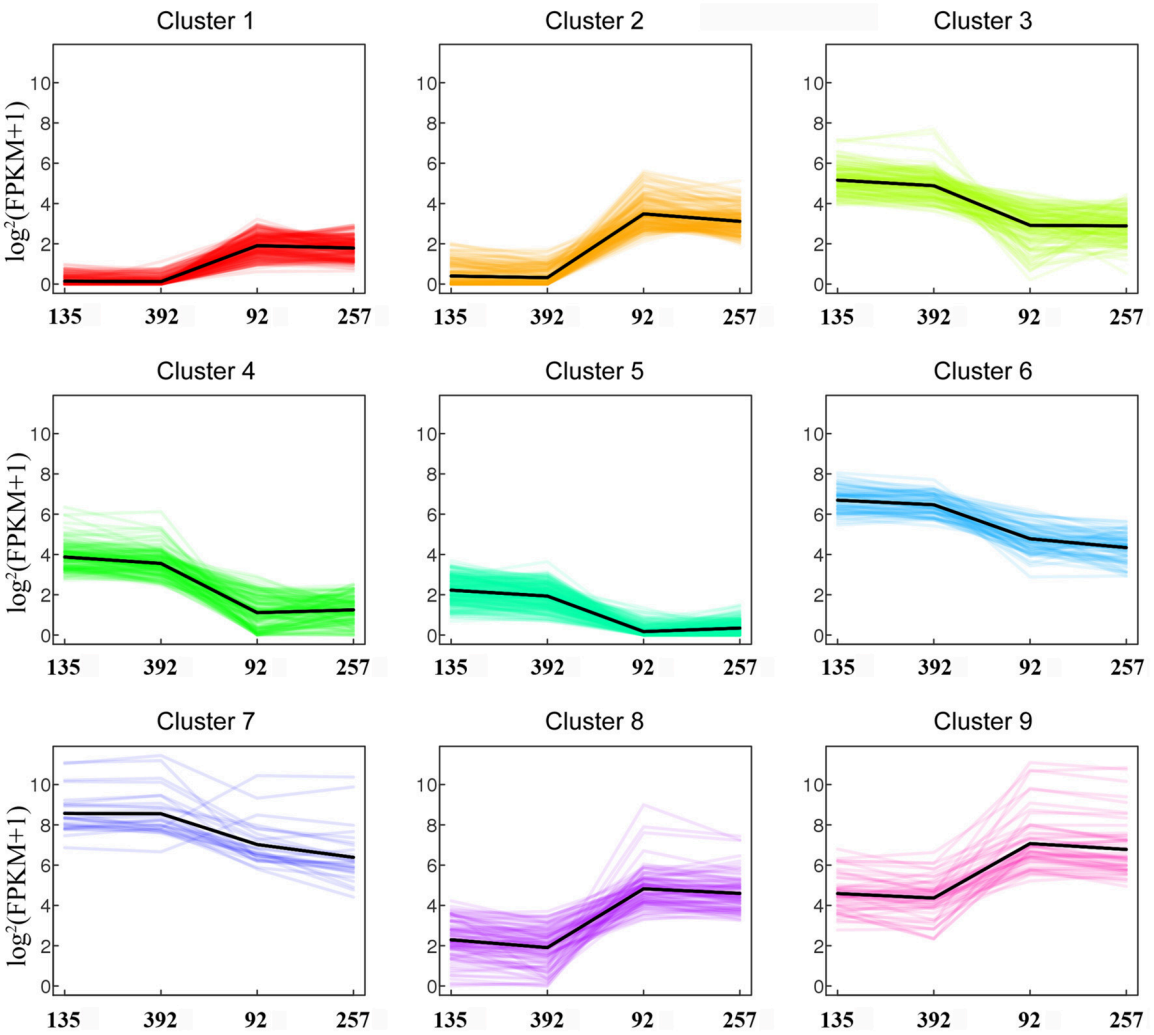
**Expression profiles of differentially expressed unigenes (DEGs)**.

Based on strict criteria, there were 1,285 unigenes that were differentially expressed between the shattering-susceptible and shattering-resistant vetch accessions, of which 575 were up-regulated and 710 were down-regulated (Figure 8A). Further analysis of the DEGs revealed that 1,087 (84.6%) genes overlapped in different types of pod shattering and that 48 (3.7%) were expressed in only shattering-resistant accessions, and 150 (11.7%) were expressed in only shattering-susceptible accessions (Figure 8B). To obtain an overview of the dependency of the changes in DEG expression, a K-means clustering analysis using MIV was performed to identify the expression patterns of differential genes associated with pod shattering in the two pod shattering types of common vetch. A total of 1,285 DEGs were clustered into nine distinct clusters with similar expression patterns, including five down-regulated (profile 3, 4, 5, 6, and 7) patterns and four up-regulated (profile 1, 2, 8, and 9) patterns (Figure 9).

### GO functional analysis of the DEGs

A total of 448 (34.86%) DEGs associated with pod shattering were divided into 51 GO categories (Figure S8). In the cellular component category, “cell part” (26.1%) was the dominant group and was followed by “membrane” (13.2%), and “organelle” (12.3%). For the molecular function category, “catalytic activity” (56.9%) and “binding” (49.1%) were the most dominant groups. Regarding the biological process category, 57.8% and 45.3% of the unigenes were assigned to “metabolic process” and “cellular process.”

### KEGG pathway enrichment analysis of the DEGs

We performed a KEGG pathway enrichment analysis on all 1,285 DEGs to understand the characteristics of the complex biological behavior of the transcriptome. A *p*-value < 0.05 was considered to indicate significant overexpression of a KEGG pathway. The enriched and downregulated unigenes in the pod ventral suture of vetch were classified into 50 functional groups. The pathways categorized as “starch and sucrose metabolism” (ko00500) and “pentose and glucuronate interconversions” (ko00040) were found to be significantly overexpressed and the number of up-regulated unigenes was significantly higher than the number of down-regulated unigenes (Figure S9). The “pectin metabolism” was included in the pathways of both ko00040 and ko00500, while the “cellulose metabolism” was only included in the ko00040 pathways. Pectin and cellulose are the main components of the plant cell wall, and its hydrolysis and metabolism have a significant effect on cell wall degradation.

### Identification of pod shattering-related genes

**Figure 10:**
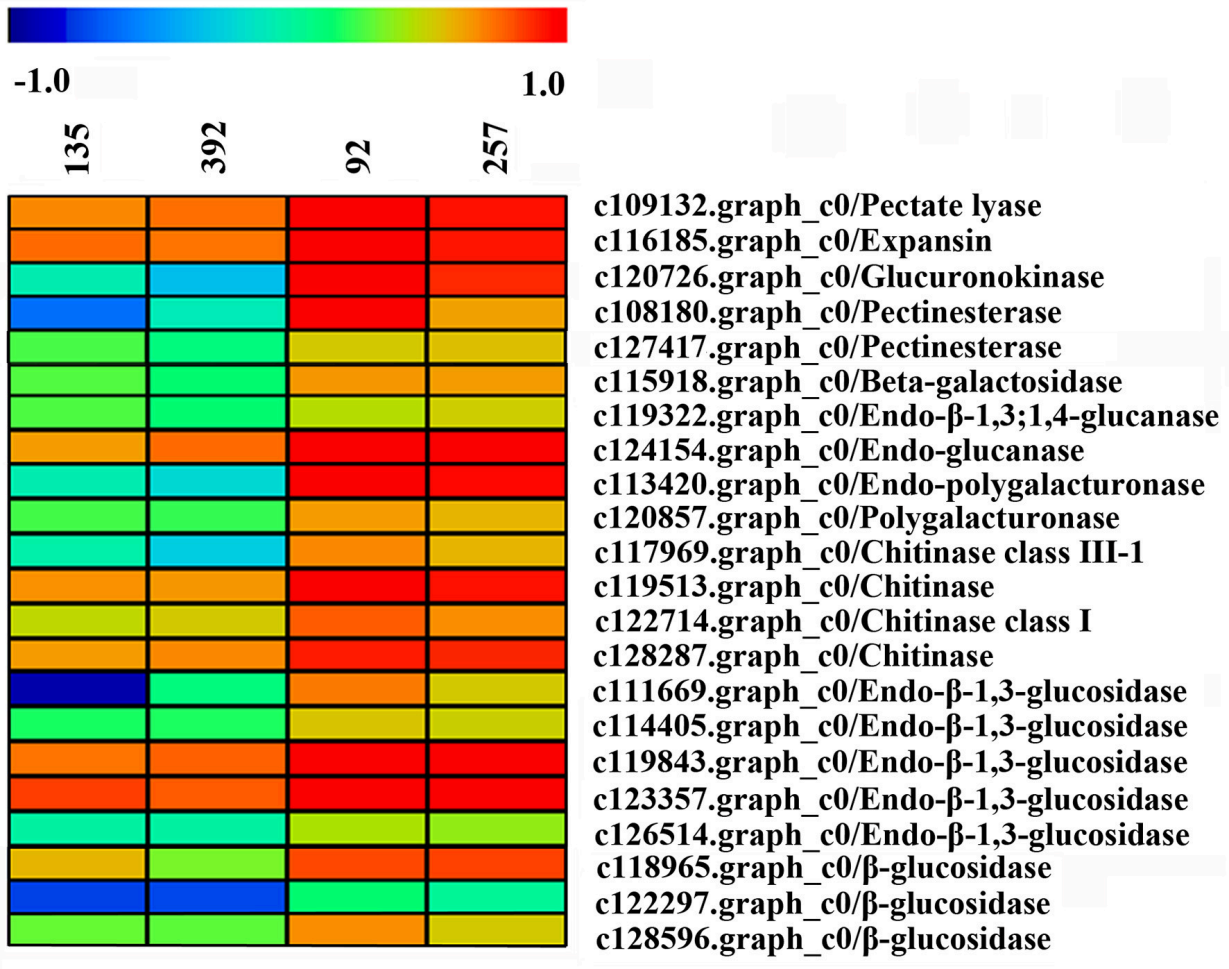
**Heat map of the expression levels of the unigenes encoding pod shattering-related proteins in pod ventral sutures of the common vetch**. The unigene expression levels are indicated with colored bars.

A large number of unigenes were obtained in this study, of which 22 DEGs were considered to be involved in pod shattering (Figure 10). Most of these unigenes encoding cell wall modification enzymes and were significantly highly expressed in the shattering-susceptible accessions. These unigenes included one pectate lyase, one β-galactosidase, one glucuronokinase, two pectinesterase, two glucanases, two polygalacturonases, four chitinases and eight glucosidases. We used agriGO to analyze the DEGs (FDR < 0.05, FC ≥ 1.3) of related pod shattering enriched in the molecular function, cellular component and biological processes terms “enrichment status” and “hierarchy.” In the unigenes that were highly expressed in the shattering-susceptible accessions, nine GO enrichment terms, such as “iron ion binding,” “hydrolase activity,” and “cationic binding” in the molecular functions category, contained known shatter-related genes (Table S5). These shatter-related hydrolases mainly included β-glucosidase, endo-β-1,3-glucosidase and endo-β-1,3;1,4-glucanase (Figure 10). Many shatter-related hydrolases highly expressed in the shattering-susceptible accessions were also predicted to be enriched in the “extracellular region,” “membrane,” and “cytoplasm” in the cellular component terms (Table S5), and were annotated as expansin, chitinase, and glucuronokinase involved in cell wall modification (Figure 10). In the biological process terms, genes involved in “oxidation reduction,” “carbohydrate metabolic process,” and “carbohydrate catabolic process” were significantly enriched and all included shatter-related genes (Table S5). These shatter-related genes were mainly included chitinase, β-galactosidase and endo-polygalacturonase (Figure 10). Among them, pectinesterase, polygalacturonase, glucuronidase, and one of the glucanases are mainly involved in pectin metabolism, whereas glucosidase and the other glucanase are mainly involved in cellulose metabolism (Figure S10). We also detected expansin and chitinase, which play important roles in cell wall decomposition. Two unigenes encoding cellulase synthase were detected, which were significantly more highly expressed in the shattering-resistant accessions (Figure S11A). Besides these previously identified pod shatter-related genes, other DEGs could also likely be involved in pod shattering in the vetch. The data of DEGs transcripts, all unigenes transcripts and the annotation of 1,285 DEGs (Table S6) have been deposited in the Harvard dataverse (doi: 10.7910/DVN/RJSEP5).

## Discussion

### Sequence quality and annotation

The Illumina platform was initially used only to analyse sequences from species with a reference genome because of its short read length. With improvements in the read length of paired-end sequencing and the development of bioinformatics and computational methods, relatively short reads can also be efficiently assembled for non-model species analysis. In addition, previous studies have shown that compared with other sequencing platforms, the Illumina system provides higher quality and longer sequences, which are well assembled and can be used for transcriptome analysis (Fu et al., 2013; Liu et al., 2016).

In this study, a total of more than 143 million raw reads were produced by using the HiSeq 2000 system, with more than 30 million raw reads from each sample. These raw reads were eventually assembled into 70,742 unigenes with an average length of 772 bp (Figure S3). The average length of the unigenes was also longer than the length of the transcriptome sequences obtained by using the same platform, such as *V. sativa* (503 bp), *V. sativa* subsp. *sativa* (331 bp), *V. sativa* subsp. *nigra* (342 bp) (Kim et al., 2015), *Litchi chinensis* (601 bp) (Li et al., 2013), and *Elymus sibiricus* (645 bp) (Zhou et al., 2016). The results showed that more than 66% of the unigenes matched with functional annotations in public databases, a value higher than previously reported for *V. sativa* (33%) (Sagar et al., 2015), *L. chinensis* (59%) (Li et al., 2013), and *Mangifera indica* (62%) (Dautt-Castro et al., 2015). In addition, the qRT-PCR results demonstrated that our sequencing was accurate and reliable. Unannotated unigenes may represent untranslated regions, non-coding RNA, mis-assembly, or a common vetch-specific gene pool (Fu et al., 2013; Liu et al., 2016). These unigenes, which represent common vetch-specific genes, should serve as a useful complement to the current vetch database.

Twenty-two DEGs were found to be involved in pectin and cellulose metabolism or cell wall modifications. These results should help researchers understand the function of the genes that cause pod shattering in vetch and may serve as a reference for studies of pod shattering in other species, and facilitate further breeding programmes aimed at producing shattering-resistant crops.

### Genes associated with pod shattering

Previous studies have shown that alterations in the cell structure of the dehiscence zone is one of the main causes of pod shattering (Dong et al., 2014). However, the breakdown of the dehiscence zone is dependent on a variety of cell wall modification enzymes and genes that cause cell structure changes, such as endo-polygalacturonase (Christiansen et al., 2002), glucosidase (Muriira et al., 2015), *SHATTERPROOF1* (*SHP1*), *SHATTERPROOF2* (*SHP2*) (Liljegren et al., 2000), *SHAT1-5* (Dong et al., 2014), and *pdh1* (Funatsuki et al., 2014). The use of QTLs mapping has been able to successfully isolate and characterize the plant structure, seed shredding and pod shattering genes in rice (*Oryza sativa*), maize (*Zea mays*) and tomato (*Lycopersicon esculentum*) (Li et al., 2006; Dong et al., 2014). Over the past 15 years, a series of genomic regions associated with soybean pod shattering have been identified by high-resolution mapping of QTLs (Yamada et al., 2009; Dong et al., 2014). *qPDH1* is a QTL for pod shattering that are fine-mapped in the 134 kb region of chromosome 16 (Suzuki et al., 2010). It was found that *SHAT1-5* associated with soybean pod shattering had hitchhiking effect on closely linked loci across 116 kb region in chromosome 16 of the soybean genome (Dong et al., 2014). Analyses of enzyme activity have also shown that there are significant differences in the pod ventral sutures between shattering-susceptible and shattering-resistant soybean varieties (Agrawal et al., 2002; Christiansen et al., 2002). It has been reported that pectin and cellulose are the main components of the plant cell wall, and their content in the cell wall significantly affects the cell structure (Jung and Park, 2013; Muriira et al., 2015). In the present study, 22 DEGs associated with cell wall modification enzymes were detected, all of which were up-regulated in the shattering-susceptible accessions. In particular, the unigenes encoding several significantly over-represented enzymes involved in the metabolism of pectin and cellulose were identified in the “starch and sucrose” (ko00500) and “pentose and glucuronate interconversion” (ko00040) metabolism pathways. These unigenes included pectinesterase (EC:3.1.1.11), polygalacturonase (EC:3.2.1.15), glucuronokinase (EC:2.7.1.43), endo-glucanase (EC:3.2.1.4), and glucosidase (EC:3.2.1.21) (Figure S10). The validation of polygalacturonase activity and cellulose (β-1,3;1,4-glucanase) activity was also consistent with the expression levels of unigenes encoding polygalacturonase and cellulose, as determined by transcriptome sequencing (Figures 4, 5, 10).

### Pectin and cellulose metabolism is associated with pod shattering

Pectin is the major component of the intercellular layer of plant cell walls. In the pectin metabolism pathway, pectin is de-esterified by pectinesterase (c108180.graph_c0 and c127417.graph_c0) hydrolysis, thus causing pectic chain esterification and production of pectate, which is further hydrolysed by polygalacturonase (c120857.graph_c0) and exo-polygalacturonase to D-galacturonate. D-galacturonate is indirectly hydrolysed to D-glucuronate, whereas D-glucuronate is further hydrolysed by glucuronokinase (c120726.graph_c0) thus, the finally generating UDP-glucose (Zhang et al., 2015) (Figure S10A). With the accumulation of endo-polygalacturonase (c113420.graph_c0) activity, the middle lamella gradually dissolves and disappears, and the modification of the cell wall becomes evident (Christiansen et al., 2002). In addition to the dissolution of the middle lamella, endo-polygalacturonase also act synergistically with glucanase (c119322.graph_c0 and c119843.graph_c0), thus weakening the primary walls of the cells in the dehiscence zone (Christiansen et al., 2002). Pectate lyase (c109132.graph_c0) is thought to involve demethylation of the pectin and thus to promote the breakdown of the cell wall middle lamella (Patterson, 2001). According to previous studies, endo-polygalacturonase breaks down the pectin network in the cell wall, whereas glucanase destroys the xyloglucan network, thereby significantly destabilizing the cell wall and, in the final phase of programmed cell death, allowing cells and protoplasts to collapse (Groover and Jones, 1999; Christiansen et al., 2002). Internal turgor pressure loss due to tonoplast rupture may cause cell and primary cell wall deformation (Christiansen et al., 2002), thereby significantly widening the gap in the dehiscence zone and causing the pod to shatter more easily.

Cellulose, a cell wall polysaccharide, is a major component of the plant cell wall structure and serves as a scaffold for binding to other plant cell wall components (Lerouxel et al., 2006; Muriira et al., 2015). Previous studies have shown that an extensive hydrogen-bonding network formed between the cellulose microfibril and the hemicellulose xyloglucan is an important load-bearing structure in the primary wall in dicots (Rose and Bennett, 1999). In cellulose metabolism, cellulose is first hydrolysed by endo-glucanase (c124154.graph_c0) and exo-cellobiohydrolase, thus producing cellobiose and 1,4-β-D-glucan. Cellobiose and 1,4-β-D-glucan are further hydrolysed by β-glucosidase (c118965.graph_c0; c122297.graph_c0; c128596.graph_c0), thereby producing β-D-glucose (Figure S10B). Interestingly, two unigenes (c115986.graph_c0 and c122597.graph_c0) involved in cellulose synthesis were detected, and their expression levels were significantly higher in shattering-resistant accessions than in shattering-susceptible accessions (Figure S11A). This result showed that the rate of cellulose synthesis was low and the cellulose metabolic rate was high in pod ventral sutures of shattering-susceptible accessions, thus further aggravating the breakdown of dehiscence zone cells. Moreover, expansin (c116185.graph_c0), a synergistic enzyme in plant cell walls, is also important for coordinated wall disassembly in plant cells (Rose and Bennett, 1999). It is possible to mediate cell wall loosening or swelling by modifying the cellulose and xyloglucan network, thereby altering the substrate availability for the hydrolase and/or transglycosylase of the host (Rose and Bennett, 1999).

### Other enzymes and functional genes associated with pod shattering

Abscission involves cell wall dissolution or cell separation, which is thought to be related to cell wall destruction through the action of chitinases (Xie et al., 2011). In *Calotropis procera*, the expression of chitinase increases with fruit dehiscence (Ibrahim et al., 2016). Furthermore, a promoter-GUS construct of the chitinase gene is strongly expressed in the floral organs of *Arabidopsis* (Patterson, 2001). In the present study, four unigenes (c117969.graph_c0; c119513.graph_c0; c122714.graph_c0; c128287.graph_c0) encoding chitinase were strongly expressed in the shattering-susceptible accessions, thus suggesting that they are also involved in the cell wall dissolution or cell separation of the pod ventral suture zone.

Other hydrolases are also involved in the decomposition of the cell wall polysaccharide matrix and network, such as β-galactosidase (c115918.graph_c0). β-Galactosidase also plays an important role in the softening of apples and Japanese pears (Li et al., 2015).

Previous studies have shown that *Lepidium appelianum* fruits cannot be opened. The anatomical structure of *L. appelianum* fruits indicates a lack a dehiscence zone, and the fruits are surrounded by a continuous loop of lignified cells (Mühlhausen et al., 2013). Mühlhausen et al. (2013) have found that *APETALA2* (*AP2*) is a negative regulator of *SHP1, SHP2*, and *RPL*, and it inhibits the overexpression of these genes in the replum and valve margin of the *L. appelianum* fruit, thereby preventing fruit shattering. However, in our study, there were no significant differences in the expression level of the unigene (c125518.graph_c0) with a sequence homologous to that of the *AP2* gene detected in the two types of accessions. Other genes associated with soybean and *Arabidopsis* pod shattering, such as *SHAT1-5, pdh1, SHP1, SHP2, IND*, and *ALC*, were also not detected as differentially expressed unigenes and were low homology with our sequencing data (Figure S11B). *SHAT1-5* (Glyma16g02200) is specifically expressed in the FCC of the soybean, while *pdh1* (Glyma16g25600) is specifically expressed in the lignin-rich inner sclerenchyma of soybean pod walls. However, their putative homologs in vetch did not differently expressed between shattering-susceptible and shattering-resistant accessions. It can be explained by the different sample tissues and different anatomy between the species. There is no FCC in the pod ventral sutures of the vetch (unpublished data, submitted to Crop Science), and the present study did not include the inner sclerenchyma tissue (Figure 6). Fruit structures are highly labile during evolution, and molecular phylogenetic data consistently show that many species with similar fruit may be only distantly related, whereas species with significantly different fruit may be very closely related (Mühlhausen et al., 2013). In addition, the sampling period of pods in this study was not consistent with that in other studies, because the developmental stages of pods are not the same among different species. Thus, our results may indicate that other genes in the common vetch may have the same function as *AP2* or other pod shattering-related genes or that there was no significant difference in the unigene expression levels, owing to inconsistencies in the sampling time. This result may aid inf further study the function of genes related to common vetch pod shattering.

## Conclusion

In this study, a unigene analysis was performed using clustering, Gene Ontology and KEGG metabolic enrichment pathways analyses, and 1,285 differentially expressed genes were identified between two shattering-susceptible accessions and two resistant accessions. A set of significantly over-represented unigenes involved in the metabolism of pectin and cellulose in the pod ventral sutures of the shattering-susceptible common vetch accessions were identified in the “starch and sucrose” and “pentose and glucuronate interconversions” metabolism pathways. In addition, we proved that a variety of cell wall hydrolases were involved in the cellular metabolism of the pod ventral suture, which promoted pod shattering. A detailed investigation of the candidate genes associated with pod shattering identified in this study would not only deepen understanding of the common vetch pod shattering mechanism but also contribute to the study of pod shattering in other plants and provide information for the molecular breeding of crops.

## Author contributions

ZL conceived the topic. RD, DD, DL, QZ, and XC performed the experiments. RD, JZ, WX, YD, and WL analyzed all data. RD wrote the manuscript. ZL and YW revised the manuscript.

### Conflict of interest statement

The authors declare that the research was conducted in the absence of any commercial or financial relationships that could be construed as a potential conflict of interest.

## Supplementary material

Figure S1 **Clean reads in the sample libraries from the four common vetch accessions**.

Figure S2 **The distribution of the contig assembly**.

Figure S3 **Length distribution of the assembled unigenes**.

Figure S4 **A species-based distribution of BLASTX matches in the NCBI non-redundant (Nr) database**.

Figure S5 **Gene Ontology (GO) classification of the assembled transcripts**.

Figure S6 **Clusters of orthologous groups (COGs) function classifications of the assembled transcripts**.

Figure S7 **Pathway assignment based on Kyoto Encyclopedia of Genes and Genomes (KEGG). (A)** Classification based on metabolism categories. **(B)** Eighteen metabolism subcategories classified by KEGG.

Figure S8 **Gene Ontology (GO) classification of the DEGs**. The unigenes were summarized in three main categories: biological process, cellular location, and molecular function.

Figure S9 **Metabolism pathway assignments of the differentially expressed unigenes (DEGs) based on the Kyoto Encyclopedia of Genes and Genomes (KEGG)**. The genes were classified into 50 functional groups. Positive and negative numbers denoted the percentages of unigenes enriched and downregulated.

Figure S10 **Simplified diagram depicting the pectin and cellulose metabolism pathways in pod ventral sutures of the common vetch. (A)** Pectin metabolism pathway. **(B)** Cellulose metabolism pathway. The red box indicates that the gene encoding the enzyme is a DEG. The coloured boxes represent the expression levels of the genes encoding the same enzyme in the four accessions.

Figure S11 **Heat map of the expression levels of unigenes encoding other pod shattering-related proteins in pod ventral sutures of the common vetch. (A)** Heat map of unigenes encoding cellulose synthase. **(B)** Heat map of unigenes encoding *AP2, SHAT1*-5, *pdh1, IND, and ALC*. Unigene expression levels are indicated with colored bars.

Table S1 **The sampling time of different accessions, the size of the mature seeds and pods, and the number of days required for the pods to reach the mature yellow stage**. The sampling time is the number of days from the day of flowering to the day of sampling. Mature seed length and width refer to the length and width of the seed when the pod reached the mature yellow stage. Mature pod length and width refer to the length and width of the pod when the pod reached the mature yellow stage. Mature yellow pod refers to the number of days required for the pods to reach the mature yellow stage.

Table S2 **Primers used for qRT-PCR analysis. Unigene 68614 was used as an internal control in the qRT-PCR analysis**.

Table S3 **BLAST analysis of the non-redundant unigenes against public databases**.

Table S4 **KEGG pathway analysis of the assembled unigenes**.

Table S5 **Gene Ontology (GO) biological process, cellular component, and molecular function term “enrichment status” of the pod ventral suture-specific differentially expressed unigenes (DEGs)**. The DEGs exhibited an FC ≥ 1.3. The FDR-adjusted *p*-values < 0.05. The P refers to the biological process. The F refers to molecular function. The C refers to cellular component. The “^*^” indicates the GO term containing the unigenes associated with the pod shattering.

Table S6 **A list of 1,285 differentially expressed unigenes (DEGs) in pod ventral sutures of shattering-susceptible and shattering-resistant vetch accessions**. No. 135 and No. 392 are shattering-susceptible accessions. No. 92 and No. 257 are shattering-resistant accessions. FPKM refers to the Fragments Per Kilobase per Million Fragments mapped. GO refers to the Gene Ontology. COG refers to the Clusters of Orthologous Groups of proteins. KEGG refers to the Kyoto Encyclopedia of Genes and Genomes. KOG refers to the euKaryotic Ortholog Groups. Swissprot refers to the Swiss-Prot protein databases. Pfam refers to the Pfam protein database. nr refers to the NCBI non-redundant protein sequences. “–” Indicates that the unigene was not successfully annotated in the corresponding database.
