# Supplementary figures and images for: Transcriptome Analyses Reveal Candidate Pod Shattering-Associated Genes Involved in the Pod Ventral Sutures of Common Vetch (Vicia sativa L.)
Source: Front Plant Sci. 2017 Apr 27;8:649. doi: 10.3389/fpls.2017.00649 (PMC5406471; doi:10.3389/fpls.2017.00649)

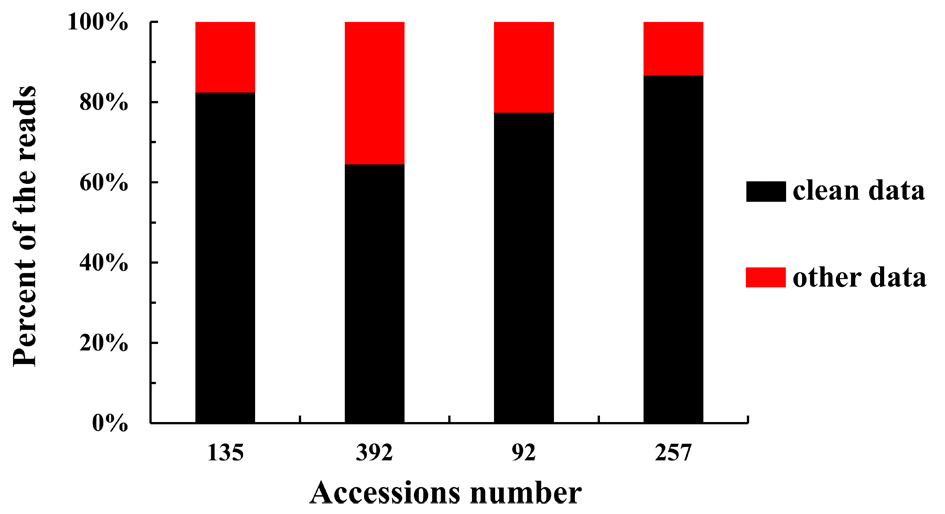

Supplement: Figure S1 — Clean reads in the sample libraries from the four common vetch accessions. [file Image1.TIF]

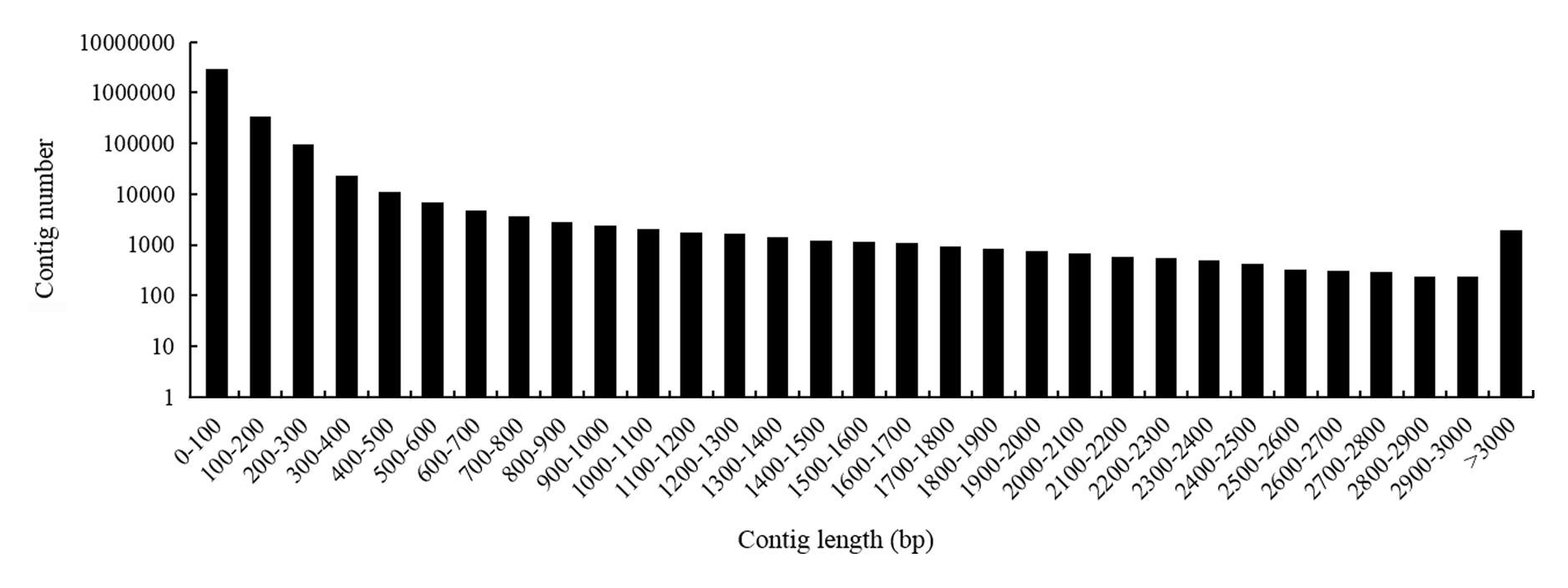

Supplement: Figure S2 — The distribution of the contig assembly. [file Image2.TIF]

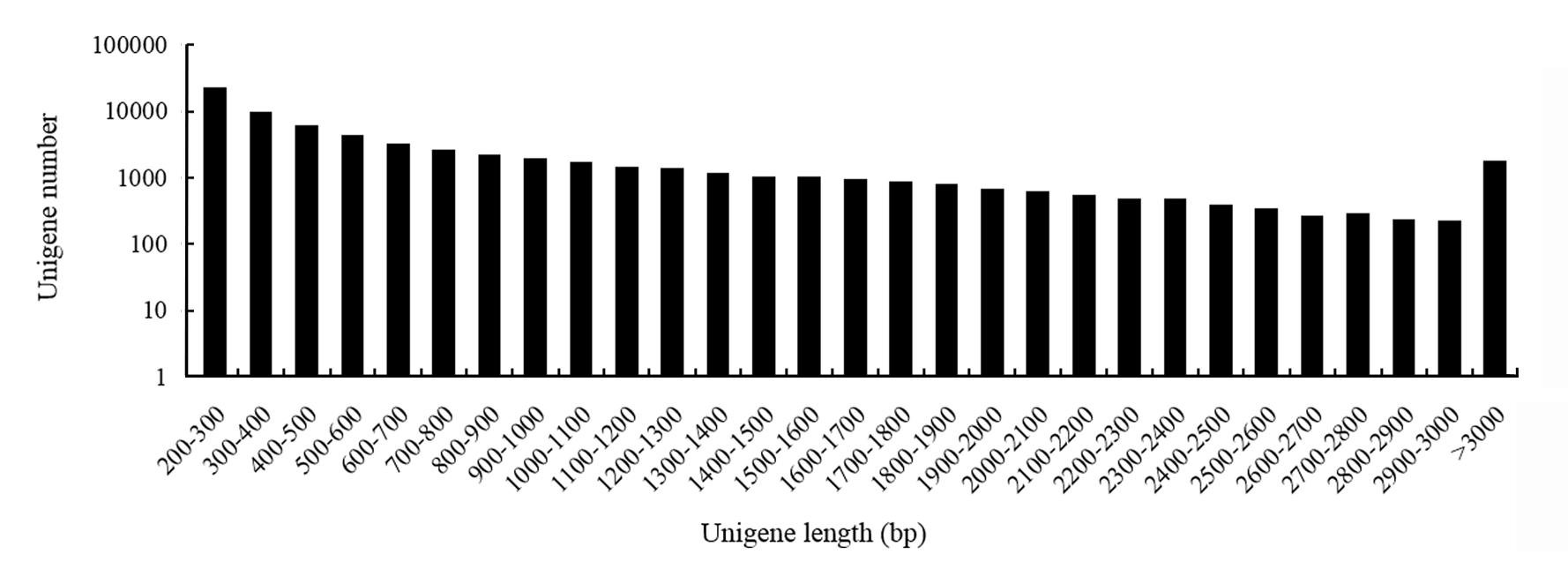

Supplement: Figure S3 — Length distribution of the assembled unigenes. [file Image3.TIF]

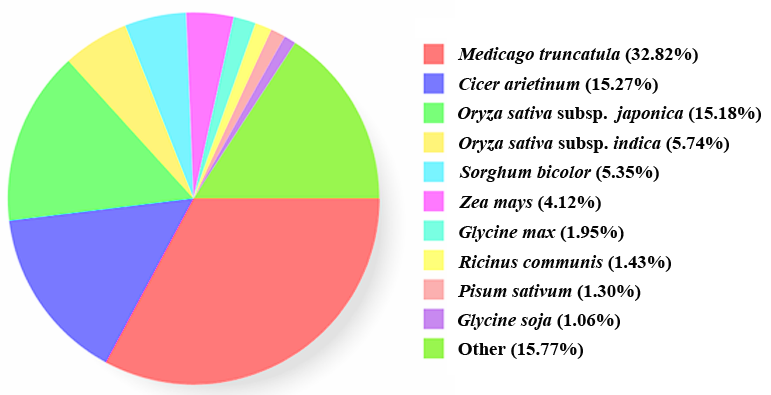

Supplement: Figure S4 — A species-based distribution of BLASTX matches in the NCBI non-redundant (Nr) database. [file Image4.TIF]

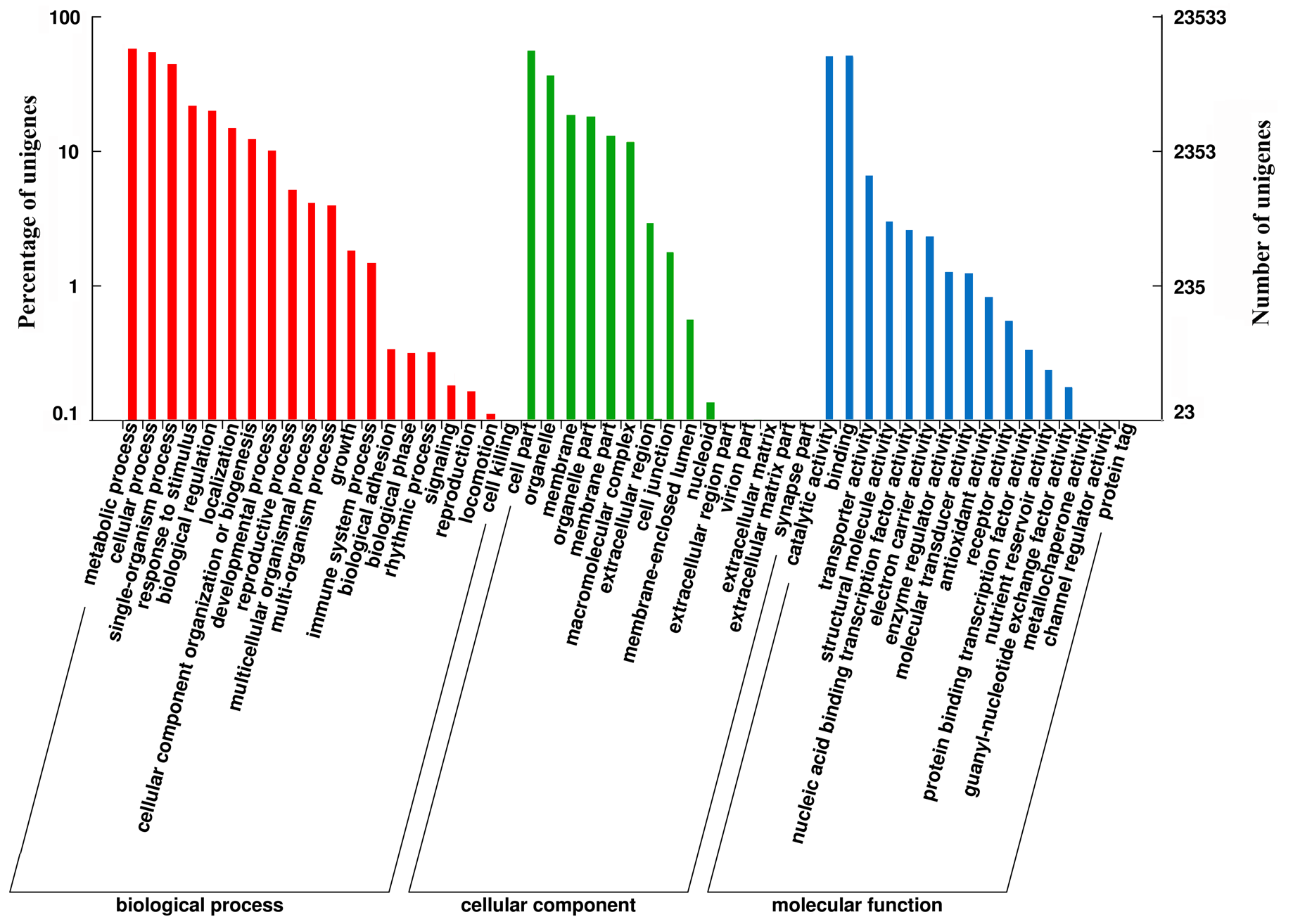

Supplement: Figure S5 — Gene Ontology (GO) classification of the assembled transcripts. [file Image5.TIF]

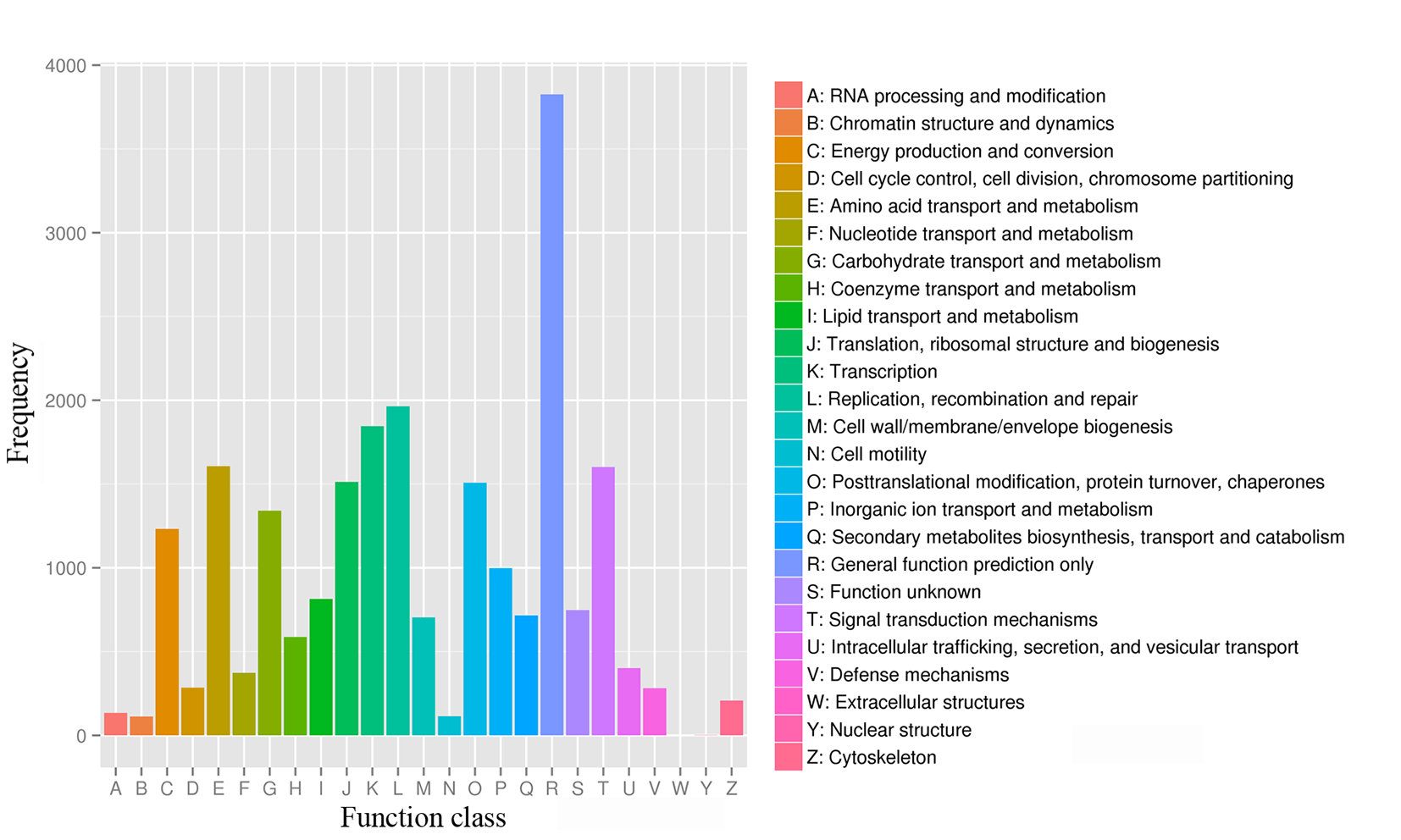

Supplement: Figure S6 — Clusters of orthologous groups (COGs) function classifications of the assembled transcripts. [file Image6.TIF]

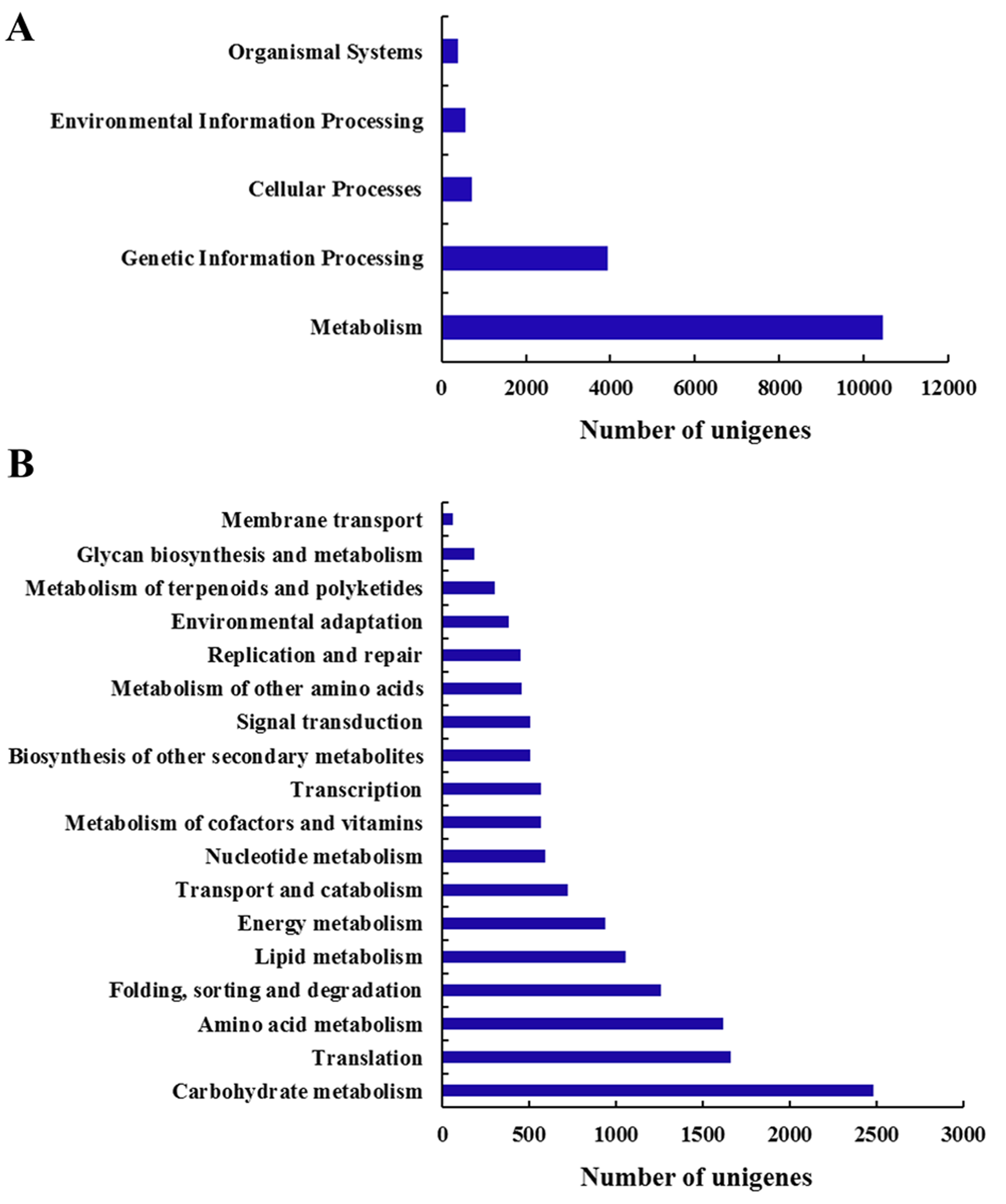

Supplement: Figure S7 — Pathway assignment based on Kyoto Encyclopedia of Genes and Genomes (KEGG). (A) Classification based on metabolism categories. (B) Eighteen metabolism subcategories classified by KEGG. [file Image7.TIF]

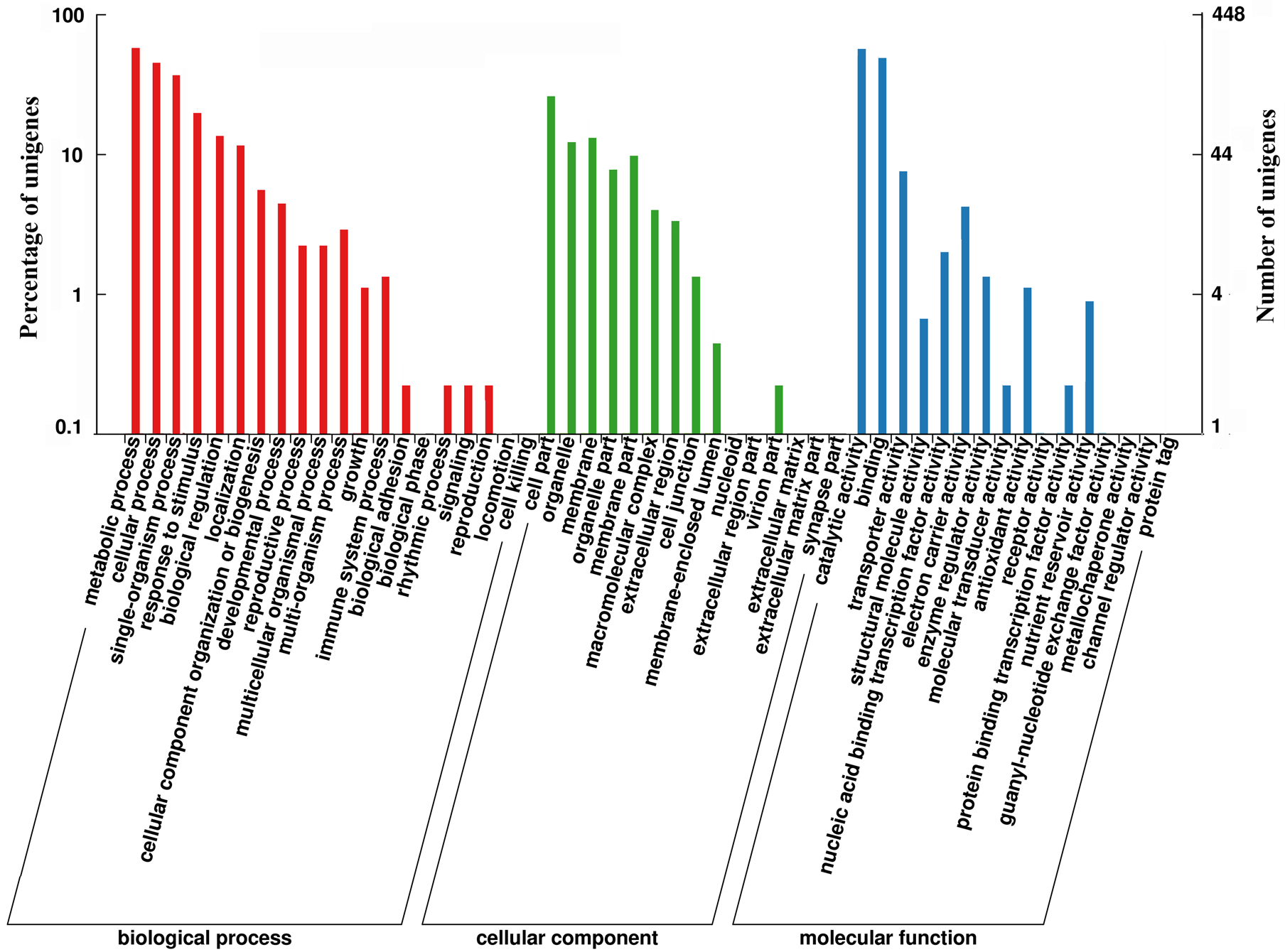

Supplement: Figure S8 — Gene Ontology (GO) classification of the DEGs. The unigenes were summarized in three main categories: biological process, cellular location, and molecular function. [file Image8.TIF]

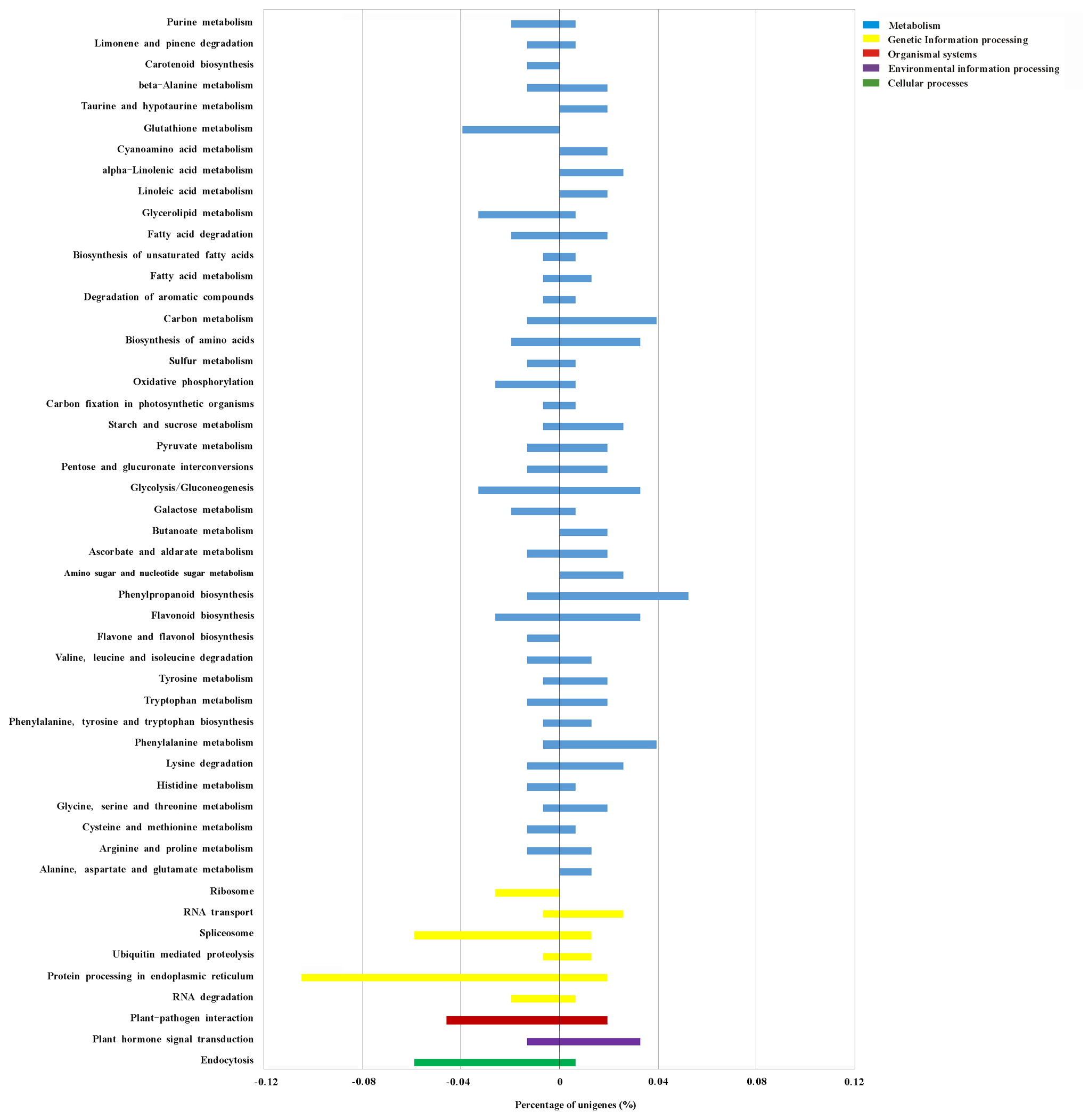

Supplement: Figure S9 — Metabolism pathway assignments of the differentially expressed unigenes (DEGs) based on the Kyoto Encyclopedia of Genes and Genomes (KEGG). The genes were classified into 50 functional groups. Positive and negative numbers denoted the percentages of unigenes enriched and downregulated. [file Image9.TIF]

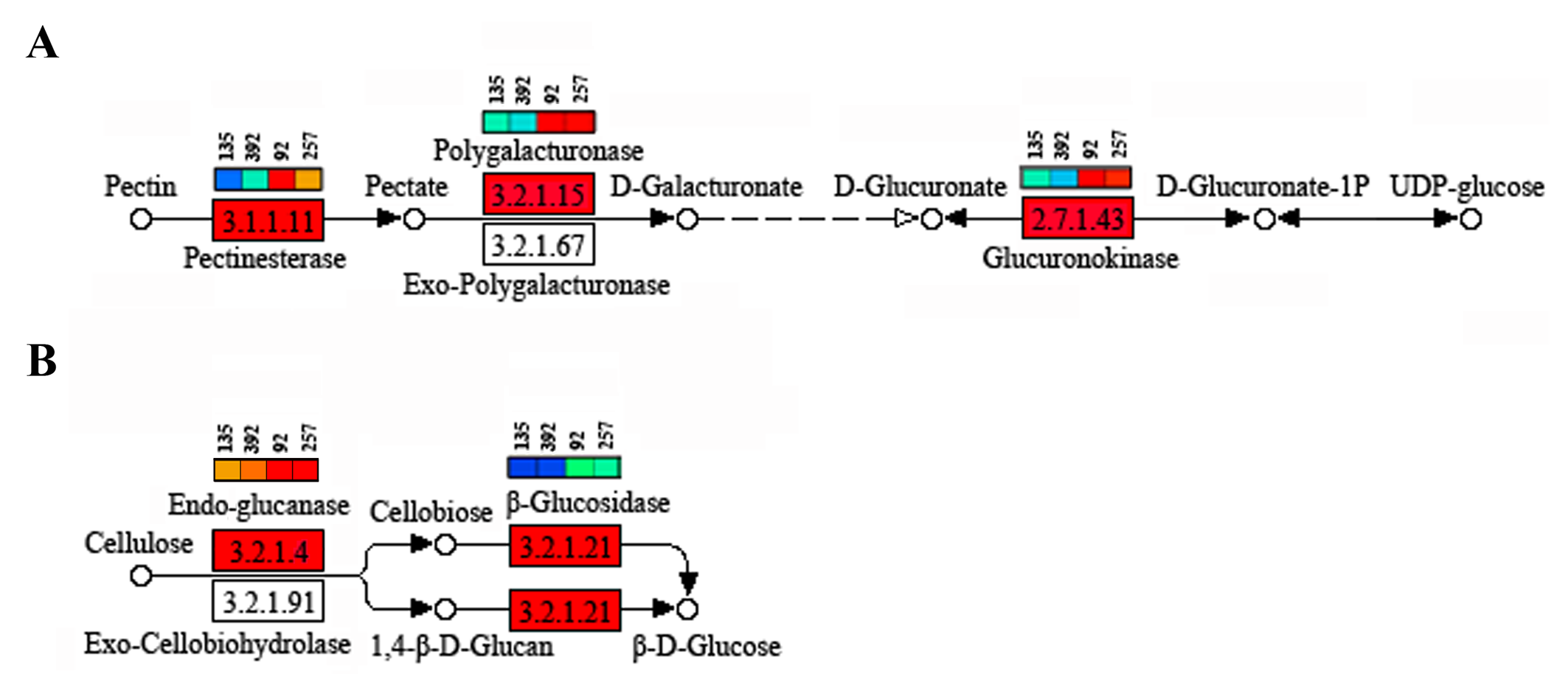

Supplement: Figure S10 — Simplified diagram depicting the pectin and cellulose metabolism pathways in pod ventral sutures of the common vetch. (A) Pectin metabolism pathway. (B) Cellulose metabolism pathway. The red box indicates that the gene encoding the enzyme is a DEG. The coloured boxes represent the expression levels of the genes encoding the same enzyme in the four accessions. [file Image10.TIF]

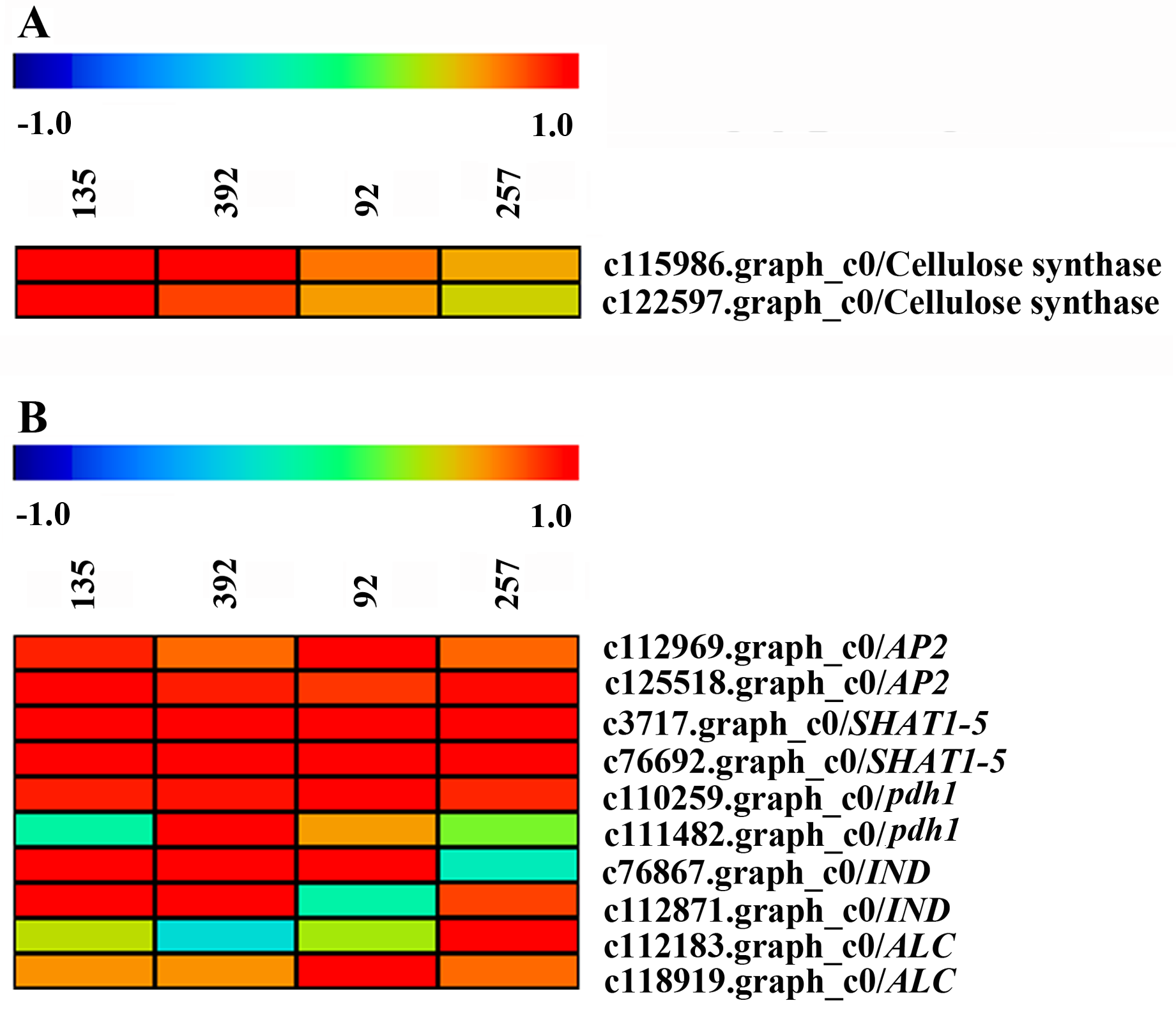

Supplement: Figure S11 — Heat map of the expression levels of unigenes encoding other pod shattering-related proteins in pod ventral sutures of the common vetch. (A) Heat map of unigenes encoding cellulose synthase. (B) Heat map of unigenes encoding AP2, SHAT1-5, pdh1, IND, and ALC. Unigene expression levels are indicated with colored bars. [file Image11.TIF]
